# Quality assessment of mHealth apps: a scoping review

**DOI:** 10.3389/frhs.2024.1372871

**Published:** 2024-05-01

**Authors:** Godwin Denk Giebel, Christian Speckemeier, Nils Frederik Schrader, Carina Abels, Felix Plescher, Vivienne Hillerich, Desiree Wiedemann, Kirstin Börchers, Jürgen Wasem, Nikola Blase, Silke Neusser

**Affiliations:** ^1^ Institute for Healthcare Management and Research, University of Duisburg-Essen, Essen, Germany; ^2^ QM BÖRCHERS CONSULTING+, Herne, Germany

**Keywords:** mHealth, quality, apps, scoping review, assessment, Digital Health Application

## Abstract

**Introduction:**

The number of mHealth apps has increased rapidly during recent years. Literature suggests a number of problems and barriers to the adoption of mHealth apps, including issues such as validity, usability, as well as data privacy and security. Continuous quality assessment and assurance systems might help to overcome these barriers. Aim of this scoping review was to collate literature on quality assessment tools and quality assurance systems for mHealth apps, compile the components of the tools, and derive overarching quality dimensions, which are potentially relevant for the continuous quality assessment of mHealth apps.

**Methods:**

Literature searches were performed in Medline, EMBASE and PsycInfo. Articles in English or German language were included if they contained information on development, application, or validation of generic concepts of quality assessment or quality assurance of mHealth apps. Screening and extraction were carried out by two researchers independently. Identified quality criteria and aspects were extracted and clustered into quality dimensions.

**Results:**

A total of 70 publications met inclusion criteria. Included publications contain information on five quality assurance systems and further 24 quality assessment tools for mHealth apps. Of these 29 systems/tools, 8 were developed for the assessment of mHealth apps for specific diseases, 16 for assessing mHealth apps for all fields of health and another five are not restricted to health apps. Identified quality criteria and aspects were extracted and grouped into a total of 14 quality dimensions, namely “information and transparency”, “validity and (added) value”, “(medical) safety”, “interoperability and compatibility”, “actuality”, “engagement”, “data privacy and data security”, “usability and design”, “technology”, “organizational aspects”, “social aspects”, “legal aspects”, “equity and equality”, and “cost(-effectiveness)”.

**Discussion:**

This scoping review provides a broad overview of existing quality assessment and assurance systems. Many of the tools included cover only a few dimensions and aspects and therefore do not allow for a comprehensive quality assessment or quality assurance. Our findings can contribute to the development of continuous quality assessment and assurance systems for mHealth apps.

**Systematic Review Registration:**

https://www.researchprotocols.org/2022/7/e36974/, International Registered Report Identifier, IRRID (DERR1-10.2196/36974).

## 1 Introduction

The number of mobile phone users in 2023 was estimated at 7.3 billion worldwide, representing over 90% of the world's population (1, 2). The intensive use of mobile devices has affected many industries, including the proliferation of mobile healthcare (mHealth) apps (3). While a universally accepted definition is lacking (4), the term mHealth is broadly defined as using “portable devices with the capability to create, store, retrieve, and transmit data in real time between end users for the purpose of improving patient safety and quality of care” (5). As an integral part of eHealth, mHealth apps aim to improve access to evidence-based information and engage patients directly in treatments by enabling providers (e.g., doctors, healthcare facilities) to connect with patients (6, 7). As such, mHealth apps have the potential to improve healthcare through accessible, effective and cost-effective interventions (8). In times of demographic change and healthcare workforce shortages, high-quality apps might contribute to sustainable healthcare (9). Especially with the rise of chronic diseases, mHealth apps can be an opportunity for prevention and improved treatment, as these diseases require constant self-care and monitoring (10). Despite this potential, literature suggests a scarcity of high-quality mHealth apps (11). In line with this, a scoping review identified several problems and barriers to the utilization of mHealth apps, including issues related to validity, usability, as well as data privacy and security, among others (12). Particularly with the widespread use of mHealth apps, it is important to avoid quality issues such as misinformation, which can limit effectiveness or potentially harm the user. As in other areas of health care, high standards are needed for evidence-based and high-quality mHealth apps (12). Appropriate quality assessment and assurance is therefore needed both during the development and ongoing use of mHealth apps.

According to a World Health Organization's definition, quality of care is “the degree to which health services for individuals and populations increase the likelihood of desired health outcomes” (13). While in general, health care quality is a multidimensional construct (14), quality dimensions in mHealth differ from those in other existing healthcare services (15). With its fast-track procedure, Germany was the first country in the world to create a system that makes selected, tested mHealth apps [called “Digital Health Applications” (DiGA)] an integral part of healthcare (16). The Federal Institute for Drugs and Medical Devices (BfArM) has set certain requirements the app must meet in order to be included in the so called “DiGA directory”. These apps have to demonstrate scientifically proven evidence of a benefit, either in the form of medical benefits or patient-relevant structure and process improvements for the patient (16). Furthermore, they must meet requirements for product safety and functionality, privacy and information security, interoperability, robustness, consumer protection, usability, provider support, medical content quality and patient safety. Once listed in the directory, patients can request these mHealth apps from their health insurance company, or the apps can be prescribed directly (16).

Currently there is a need for further adjustments to the fast-track procedure on the part of providers, health insurers and manufacturers (9). For example, the National Association of Statutory Health Insurance Funds (GKV-Spitzenverband) requires, among other things, that quality specifications must be met for user-friendly and target group-oriented design, data protection and data security (17).

*Quality assessment tools:* In addition to country-specific approaches, a number of simple assessment tools have been developed, such as the Mobile App Rating Scale (MARS) (18), ENLIGHT (19) or the System Usability Scale (SUS) (20). These approaches (in the following called *quality assessment tools*) typically assess the quality of apps with a number of items and provide the user with a score. For example, as one of the most widely used evaluation tools, MARS was developed on the basis of a literature review of existing criteria for evaluating the quality of apps and subsequent categorization by a panel of experts. The resulting multidimensional rating scale covers the areas of engagement, functionality, aesthetics, information and subjective quality of apps. Resulting scores are intended to be used by researchers, guide app developers, or to inform health professionals and policymakers (18, 19).

*Quality assurance systems:* In addition, approaches have been developed (hereinafter referred to as *quality assurance systems*) which go beyond traditional scoring instruments, e.g., by providing a framework for assessing the mHealth apps along their product lifecycle. For example, Sadegh et al. (21) propose an mHealth evaluation framework through three different stages of the app's lifecycle. Similarly, Mathews et al. (22) detail a framework assessing technical, clinical, usability, and cost aspects pre- and post-market entry. To date, there is no overview in the literature that differentiates between quality assessment tools and quality assurance systems.

Therefore, and in view of the situation in Germany described above, this work pursues two objectives: (1) to collate literature on quality assessment tools and quality assurance systems for mHealth apps, compile the components of the tools, and group them into overarching quality dimensions, which are potentially relevant for the continuous quality assessment of mHealth apps; (2) to identify and characterize quality assurance systems with a view to continuous quality assurance.

Relevant information can be extracted from publications in which the tools are developed or validated. Studies in which the tools are used for the evaluation of apps are also potentially relevant, as they provide evidence that the respective tools have been applied for the assessment of an mHealth app by researchers. The method of scoping review was found feasible, as no single precise question regarding feasibility, appropriateness, meaningfulness or effectiveness had to be answered (23). While specific questions of effectiveness are traditionally answered by collating quantitative literature in a systematic review, scoping reviews are used to map literature and address a broader research question (e.g., identify gaps in research, clarify concepts, or report on types of evidence that inform clinical practice) (24).

This work is part of a larger research project (QuaSiApps—Ongoing Quality Assurance of Health Apps Used in Statutory Health Insurance Care), which is funded by the Innovation Fund of the Federal Joint Committee and aims to create a concept for continuous quality assurance of mHealth apps.

## 2 Methods

A scoping review was conducted to answer the following questions: Which quality assessment tools and quality assurance systems have been developed and/or used in the field of mHealth apps? Which items do they consist of? Which quality dimensions can be derived from the quality assessment tools and quality assurance systems? To answer these questions, we followed the key phases outlined by Levac et al. (25), including identifying relevant studies, study selection, charting the data, and collating, summarizing, and reporting the results. Reporting followed the PRISMA extension for scoping reviews (26). A review protocol was written and published prior to screening (27). The protocol contains detailed information on the databases searched, the search terms used, and the inclusion and exclusion criteria applied during the screening process.

### 2.1 Literature search

The electronic indexed databases Medline, EMBASE and PsycInfo were searched for primary literature on the topic. Studies containing description of a literature review were included, if the review served to develop the items of the assessment tool presented. However, the focus had to be on the development and description of a specific tool. Search strategies were developed through discussion (GG, NS, CS) and with aid of the working group leader (SN). The strategies were pilot tested and refined. The search strategies comprise of keywords and synonyms for assessment tools and mHealth. All bibliographic searches were adapted to the databases’ requirements. Full search strategies and number of hits per keyword can be found in the review protocol. Searches were executed on July 26th, 2021. Reference lists of included articles were screened for further eligible literature. Further information such as the search string can be found in the corresponding research protocol (27).

### 2.2 Inclusion and exclusion criteria

Studies were eligible for inclusion if they fulfilled the following criteria: (1) included either development, or description, or further information on disease-independent concepts of quality assessment or quality assurance of mHealth apps, (2) were in English or German language, and (3) were published between January 1st, 2016 and July 26th, 2021. This means that studies were included if quality assessment tools and quality assurance systems were applied (application studies), developed (development studies) or validated (validation studies). In order to incorporate approaches currently in use, the quality assessment tools and quality assurance systems used in application and validation studies were identified and included, even if they were published before January 1st, 2016. For application studies, the investigated mHealth apps had to be used by patients in outpatient treatment and needed to have more functions than improvement of adherence, text-messaging, reminder or screening for primary prevention or (video) consultation or disease education or reading out and controlling of devices.

Applied exclusion criteria were: (1) articles that did not include information on quality assessment tools or quality assurance systems, (2) the investigated quality assessment or quality assurance system was not disease-independent, (3) the assessed mHealth app had not more functions than the following: improvement of adherence, text-messaging, reminder or screening for primary prevention or (video) consultation or disease education or reading out and controlling of devices, (4) The mHealth app evaluated was not primarily for patient use, (5) the assessed mHealth app is not used in outpatient treatment, (6) articles that included only research protocols, conference abstracts, letters to the editor, or expression of opinions. Apart from the publication date, the inclusion and exclusion criteria were all set manually and not using the filter function of the databases. Further information on the inclusion and exclusion criteria, including the search timeframe, can be found in the review protocol (27).

### 2.3 Selection of relevant studies

Identified results were loaded into the EndNote reference management program (Clarivate Analytics, Philadelphia, US; version X9). Duplicates were removed automatically and manually during the screening process. All unique references were screened in terms of their potential relevance based on title and abstract. Documents considered potentially relevant were reviewed in full-text and retained if the study met inclusion criteria. Two researchers (GG, NS) performed all screening steps independently. Any disagreements were resolved by consulting a senior researcher (SN).

### 2.4 Extraction and analysis of data

Included studies were extracted in tables by two persons independently (GG, CS). Relevant data of included articles was marked and extracted using MAXQDA 2022 (Verbi Software GmbH, Berlin). In a first step, articles were categorized into application studies, validation studies, and development studies and were then extracted into pre-specified tables. The extraction table for application studies comprised author(s), year, country, the used quality assessment tool(s), investigated disease(s) or the field(s) of application, the number of investigated apps, the study type and the source of the tool used in the application study. Data extraction from validation studies included author(s), year, country, the validate quality assessment tool(s), investigated disease(s)/field(s) of application and the origin of the validated tool. The extraction table for development studies included author(s), year, country, quality assessment tool, disease(s)/field(s) of application, the quality dimensions described and named by the author of the respective studies and the attribution to the quality dimensions developed in this scoping review.

Identified items were extracted and grouped into clusters in Microsoft Excel by one researcher (GG) and quality-checked by two researchers (FP, CA). Results were compared and discussed in case of disagreement. If necessary, a senior researcher was involved (SN). In case a criterion or aspect did not match into an existing cluster, a new cluster was created. Based on the information from the literature analyzed, the clusters were labeled. The labeled clusters were described and constituted the quality dimensions. The results were summarized, systemized and presented in tables.

## 3 Results

**Figure 1:**
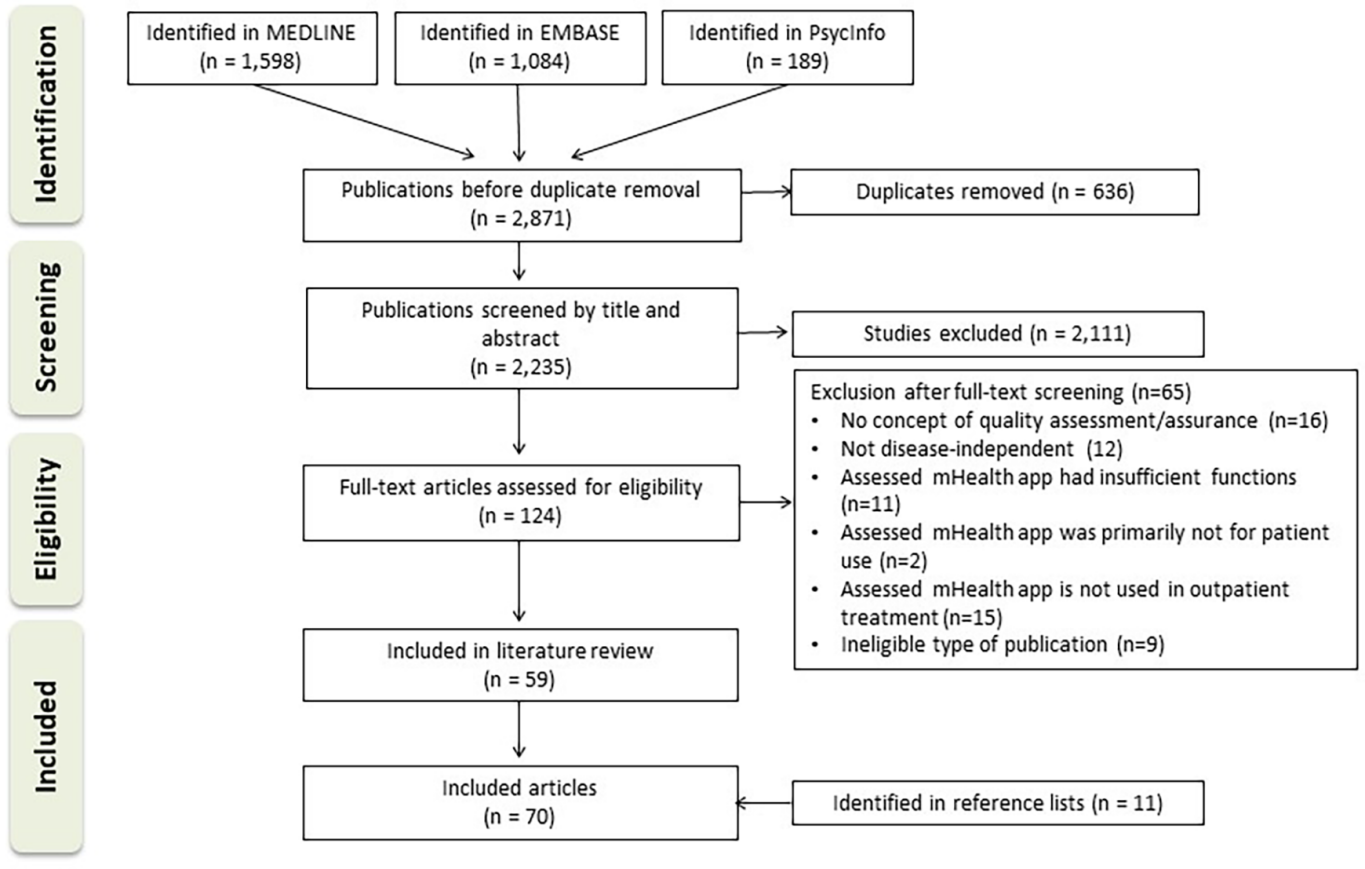
Flow diagram depicting the selection of sources of evidence.

The selection process is shown in Figure 1. A total of 2,871 articles were identified in the three databases. Of these, 2,235 articles remained after duplicate removal and were screened according to title and abstract. One hundred and twenty-four articles were included in full-text screening and subsequently, 59 studies met inclusion criteria. See Supplementary Appendix A for a table of studies excluded in full-text screening. A further 11 articles were identified via citation searching. This refers to the studies in which the tools mentioned in the application studies were developed. In total, 70 articles were included.

**Figure 2:**
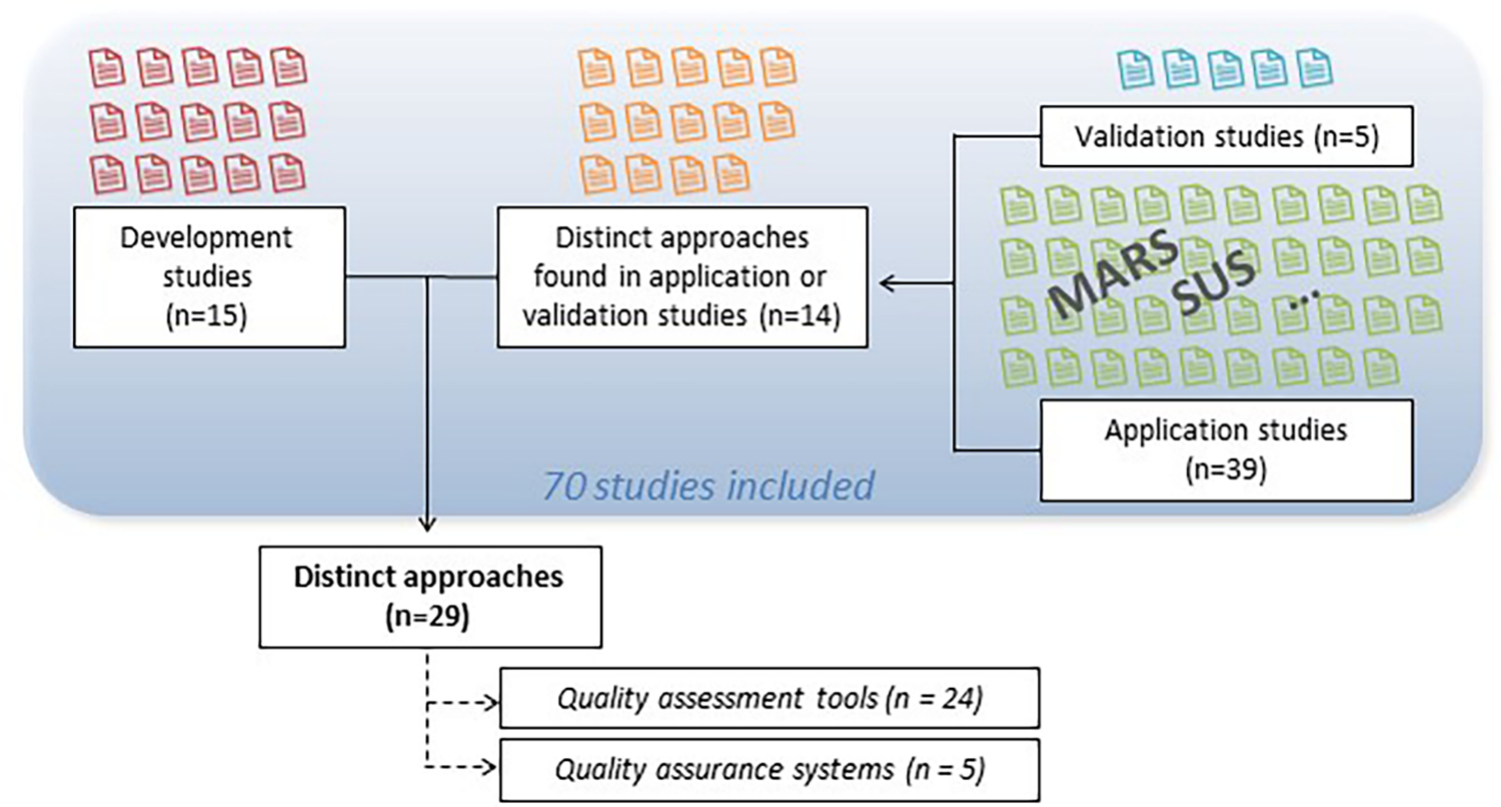
Included studies and approaches.

In 15 of the included articles, a quality assurance system or a quality assessment tool was developed (10, 21, 28–40). Five of the included articles were validation studies (8, 41–44). In addition to development and validation studies, a number of studies (*n* = 39) were identified in which quality assessment tools were applied. Of these, 19 studies employed the Mobile App Rating Scale (MARS) which was developed by Stoyanov et al. (18) or a modified version of it (33, 38, 45–61), and one study (62) used the user version of the MARS (uMARS) proposed by Stoyanov et al. (63). Another ten studies (39, 64–72) employed the System Usability Scale (SUS) developed by Brooke et al. (20). Two studies (73–75) used the (modified) Silberg scale. Further seven studies were identified in which additional quality assessment tools and quality assurance systems were applied (76–82). In total, 14 quality assurance systems and quality assessment tools were found in application or validation studies (18–20, 22, 63, 73, 83–90). Of note, the articles by Liu et al. (33), Tan et al. (38) and Wood et al. (39) report on both development and application and were therefore classified in both categories. An overview of all included articles is given in Supplementary Appendix B. In the 74 included articles, a total of 29 distinct approaches to quality assurance or quality assessment were identified. Five of these have been identified as quality assurance systems (21, 22, 30, 35, 90), while the remaining 24 tools are considered quality assessment tools. Figure 2 gives an overview of the different types of studies included.

### 3.1 Characteristics of included quality assessment tools

An overview of the identified 24 quality assessment tools is presented in Table 1. Of these, 8 were developed for the assessment of mHealth apps for specific diseases or disease areas and 11 for assessing mHealth apps for all fields of health. Another five are not restricted to health apps, but have been included as they have been used for the assessment of health apps in application studies. Included articles dealing with quality assessment tools predominantly stem from the USA (*n* = 8), Australia (*n* = 4), and the UK (*n* = 4). The identified quality assessment tools were developed in different ways, e.g., by adapting existing measures, based on findings from literature and guideline review, by conducting focus groups or by mixed-methods approaches. The quality assessment tools were developed for utilization developers, academics, healthcare providers, government officials and users.

**Table 1:** Characteristics of the included quality assessment tools.

Author (year)	Country	Tool name	Field of application
Baumel et al. (2017) (19)	US	ENLIGHT	All fields of health
Berry et al. (2018) (28)	UK	Mobile Agnew Relationship Measure (mARM) Questionnaire	Mental health
Brooke et al. (1996) (20)	UK	System Usability Scale (SUS)	Not restricted to health
Brown et al. (2013) (29)	US	Health-ITUEM	All fields of health
Doak et al. (1996) (83)	US	Suitability Assessment of Materials (SAM)	All fields of health
Glattacker et al. (2020) (31)	Germany	Usability questionnaire	Allergic Rhinitis
Huang et al. (2020) (32)	Singapore	App-HONcode	Medication Management in Diabetes
Huckvale et al. (2015) (84)	UK	Untitled	All fields of health
Jusob et al. (2022) (10)	UK	Untitled	Chronic diseases
Lewis et al. (1995) (85)	US	After-Scenario Questionnaire (ASQ), Post-Study System Usability Questionnaire (PSSUQ), Computer System Usability Questionnaire (CSUQ)	Not restricted to health
Liu et al. (2021) (33)	China	Untitled	Traditional Chinese Medicine and Modern Medicine
Llorens-Vernet and Miro (2020) (40)	Spain	Mobile App Development and Assessment Guide (MAG)	All fields of health
Minge and Riedel (2013) (34)	Germany	meCUE	Not restricted to health
O'Rourke et al. (2020) (36)	Austria	App Quality Assessment Tool for Health-Related Apps (AQUA)	All fields of health
Pifarre et al. (2017) (37)	Spain	Untitled	Tobacco-quitting
Reichheld (2004) (86)	US	Net Promoter Score (NPS)	Not restricted to health
Ryu and Smith-Jackson (2006) (87)	US	Mobile Phone Usability Questionnaire (MPUQ)	Not restricted to health
Schnall et al. (2018) (88)	US	Health Information Technology Usability Evaluation Scale (Health-ITUES)	All fields of health
Shoemaker et al. (2014) (89)	US	Patient Education Materials Assessment Tool (PEMAT)	All fields of health
Silberg et al. (1997) (73)	Sweden	Silberg Scale	All fields of health
Stoyanov et al. (2015) (18)	Australia	Mobile App Rating Scale (MARS)	All fields of health
Stoyanov et al. (2016) (63)	Australia	User Version of the Mobile Application Rating Scale (uMARS)	All fields of health
Tan et al. (2020) (38)	Australia	Untitled	Allergic Rhinitis and/or asthma
Wood et al. (2018) (37)	Australia	Untitled	Cystic fibrosis

None of the 24 articles includes a definition of a concept for quality. Of note, Brooke et al. (20) define the concept of usability in the context of the SUS. The tools are diverse with regard to their extent. Some tools consider single aspects, such as engagement (28, 86). For example, the net promoter score (86), which was used by de Batlle et al. (64), consists of only one question. In contrast, other tools cover a wider range of aspects (19, 40).

### 3.2 Characteristics of included quality assurance systems

The identified approaches were assigned to quality assurance systems if they assessed the apps over time. An overview of the included quality assurance systems is presented in Table 2.

**Table 2:** Characteristics of included quality assurance systems.

Author (year)	Country	Tool name	Field of application
Camacho et al. (2020) (30)	US	Technology Evaluation and Assessment Criteria for Health Apps (TEACH-Apps)	All fields of health
Mathews et al. (2019) (22)	US	Digital Health Scorecard	All fields of health
Moshi et al. (2020) (35)	Australia	Health technology assessment module	All fields of health
Sadegh et al. (2018) (21)	Iran	Untitled	All fields of health
Yasini et al. (2016) (90)	France	Multidimensional assessment program	All fields of health

The five included quality assurance systems stem from the US (*n* = 2), Australia (*n* = 1), Iran (*n* = 1), and France (*n* = 1). Similar to the quality assessment tools identified, none of the five articles includes a definition of a concept for quality. The quality assurance systems were developed to be used by developers, health practitioners, government officials, and users. Camacho et al. (30) tailored an existing implementation framework and developed a process to assist stakeholders, clinicians, and users with the implementation of mobile health technology. The Technology Evaluation and Assessment Criteria for Health apps (TEACH-apps) consists of the four parts (1) preconditions, (2) preimplementation, (3) implementation, and (4) maintenance and evolution. The authors recommend to repeat the process at least biannually, in order to adapt for changing consumer preferences over time (30). Mathews et al. (22) propose a digital health scorecard consisting of four domains (technical, clinical, usability, cost), which aims to serve as framework guiding the evolution and successful delivery of validated mHealth apps over the product's lifecycle. Moshi et al. (35) have developed criteria for evaluation of mHealth apps within health technology assessment (HTA) frameworks. The multidimensional module also contains items allowing for post-market surveillance. Sadegh et al. (21) have conducted an mHealth evaluation framework throughout the lifecycle in three stages, namely (1) service requirement analysis, (2) service development, and (3) service delivery. Finally, Yasini et al. (90) have developed a multidimensional scale for quality assessment of mHealth apps.

### 3.3 Derived quality dimensions

**Figure 3:**
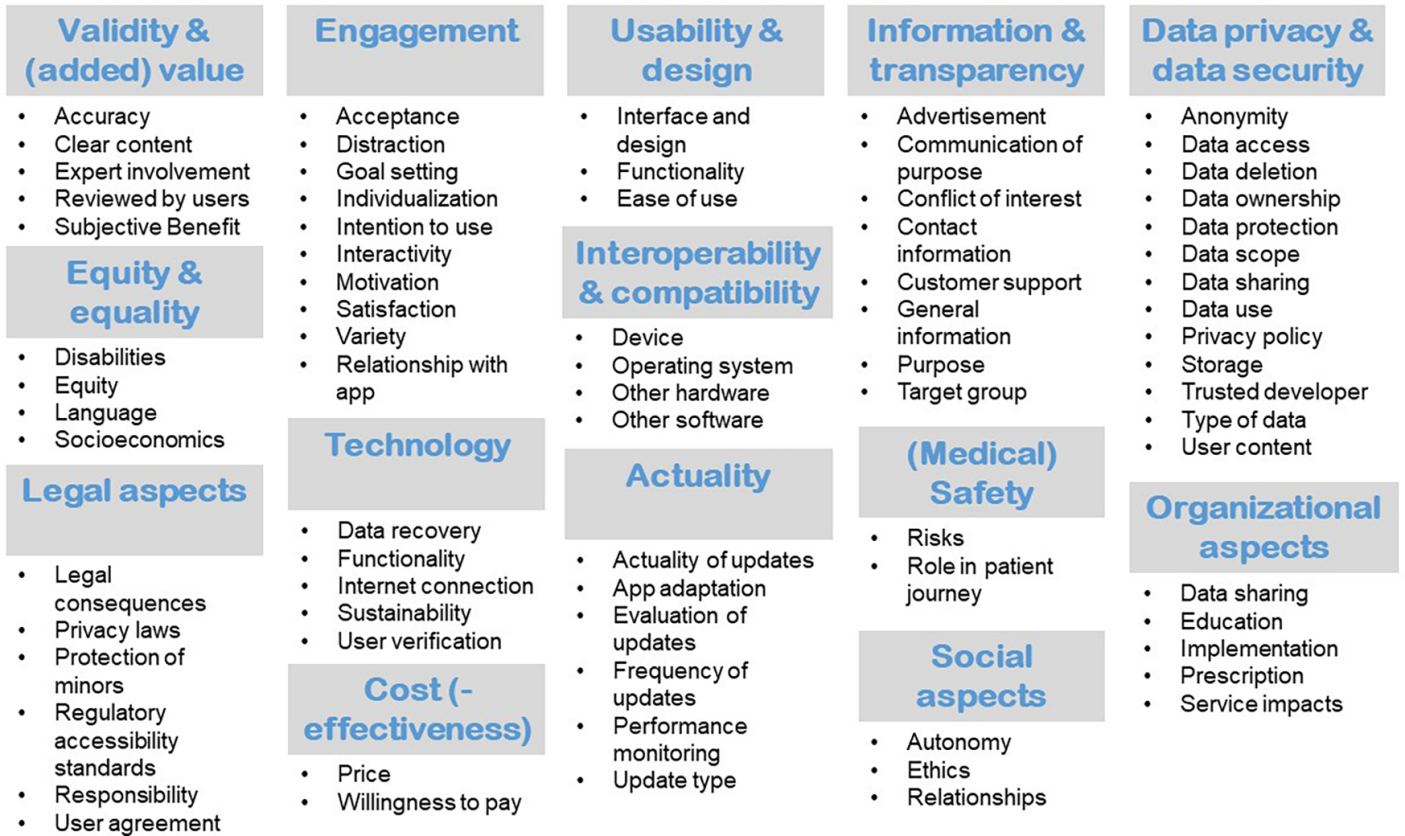
Quality dimensions with corresponding criteria.

In total, 584 items were extracted from the identified quality assessment tools and quality assurance systems and were categorized into clusters, respectively the quality dimensions. The number of quality dimensions derived from each of the 29 articles ranged from one to 13 dimensions, with an average of 4.9 dimensions. These were grouped to a total of 14 distinct quality dimensions. Figure 3 gives an overview of the identified quality dimensions and quality aspects.

Items pertaining to the quality dimension “validity and (added) value” were contained in 21 of the included quality assessment tools and quality assurance systems. Items addressing the clear, complete and accurate presentation of relevant and useful content based on evidence-based information were included here. In addition, items concerned with the provision of information about the (scientific) sources used, the involvement of experts in the development and evaluation process and the patient-specific benefits were considered relevant for this quality dimension. Twenty-one articles contained items which were grouped to “usability and design”. Usability provides information on how difficult / complex it is to operate and use the app. Usability can be indicated by ease of use. Both direct and long-term use should be taken into account. The design includes the presentation and associated clarity. The application itself, but also the results provided, should be clear and concise. Integrated functions should always be fit for purpose. The usability should be tested by usage tests before publication.

Eighteen of the 32 articles included information which was grouped to the quality dimension “engagement”. Engagement describes the user's involvement and can be indicated by the extent of use or the intention to use the app long term. It can be strengthened by calls to action, the setting of goals and human attributes such as friendliness, trust, and acceptance. Users can be motivated by interactions, personalization, interesting content and resulting fun during use. In contrast to the intention to use, the subjective benefit is not part of this dimension but belongs to Validity & (Added) Value.

**Figure 4:**
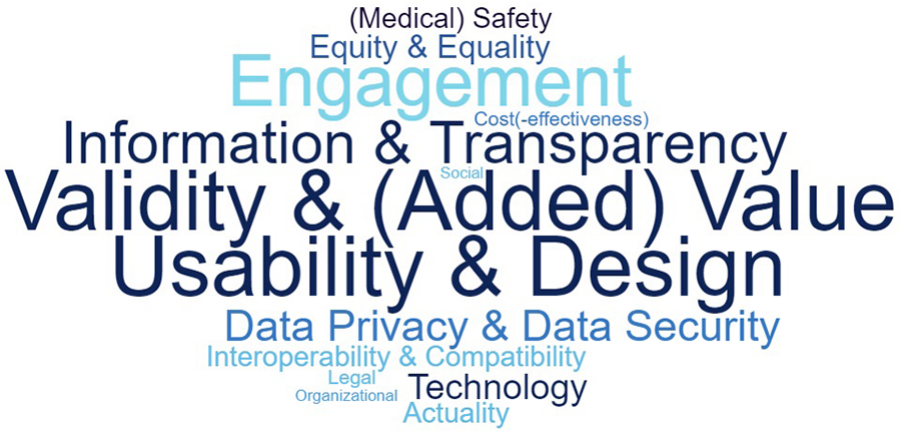
Word cloud including the quality dimensions.

Fifteen of the included articles describing quality assessment tools and quality assurance systems contained dimensions which were sorted into “information and transparency”. The dimension “data privacy and data security” was contained in 11 articles. Further dimensions are “technology” (*n* = 9), “equity and equality” (*n* = 8), “interoperability and compatibility” (*n* = 7), “(medical) safety” (*n* = 7), “actuality” (*n* = 7), “legal aspects” (*n* = 5), “(cost-)effectiveness” (*n* = 5), “social aspects” (*n* = 4), and “organizational aspects” (*n* = 4). An overview of the quality dimensions derived from the included studies can be found in Supplementary Appendix C. The full descriptions of these dimensions, which were developed based on the extracted criteria from the quality assessment tools and quality assurance systems, can be found in Supplementary Appendix D. The frequency of the individual quality dimensions in the 33 quality assessment tools and quality assurance systems included is illustrated in Figure 4.

## 4 Discussion

The aim of this scoping review was to identify relevant quality dimensions by searching and analyzing quality assessment tools and quality assurance systems. Thereby, the aim was not to obtain a complete survey of all available quality assessment tools. Such a list was meanwhile provided by Hajesmaeel-Gohari et al. (91). A total of 70 articles were included in the review, of which 29 articles contained distinct approaches of quality assessment tools and quality assurance systems.

Of the identified approaches, some include one or two aspects, while others allow a more comprehensive assessment. For example, the Net Promoter Score (86), which de Batlle et al. (64) used alongside the SUS to evaluate an mHealth-enabled integrated care model, consists of just one item. The NPS is based on the question „How likely is it that you would recommend [name of company/product/website/services] to a friend or colleague?”. In their study, de Batlle et al. (64) used the NPS to measure acceptability and thus, the score was assorted to the quality dimension “engagement” in our scoping review.

A large number of approaches from application studies were identified. In these studies, the MARS or a modified version of the MARS (*n* = 19) and the SUS (*n* = 10) were used most frequently. The MARS is a 23-item questionnaire with questions on engagement, functionality, aesthetics, the quality of the information contained and general questions for the subjective assessment of the app (18). The MARS was developed to enable a multidimensional assessment of app quality by researchers, developers, and health-professionals. The items contained in the MARS were assigned to six quality dimensions in this review, namely “information and transparency”, “validity and (added) value”, “engagement”, “usability and design”, “technology”, and “equity and equality”. The SUS, which is also frequently used, was developed by Brooke et al. in 1996 (20) with the intention of providing a “quick and dirty” tool for measuring the usability in industrial systems evaluation. It consists of ten elements, which were grouped into the dimensions “engagement” and “usability and design” in this review.

Many quality assessment tools contain questions that are easy to answer, but which reflect opinions rather than facts. For example, the items “I feel critical or disappointed in the app.” (28) or “Visual appeal: How good does the app look?” (18) can be answered in subjective ways by different people. The tools therefore consist of parameters that are used to approximate the quality of the app. In this context, it is questionable how the quality of an app can be fully measured. This may also include the consideration of problems with the app.

Many of the approaches identified focus on usability. Presumably because usability is quite easy to measure and provides app developers with important insights. For example, patient safety is rarely addressed in the identified studies. Thus, the frequency of items in different questionnaires does not necessarily indicate their relevance to the healthcare system. This could be due to the target group of the approach and the complexity of the survey. However, in the next step, it is necessary to determine which aspects are relevant to the quality of apps from the perspective of the healthcare system.

Besides the quality assessment tools and given the objective of QuaSiApps (to develop a continuous quality assurance system), a particular focus of this scoping review was to identify approaches which consider mHealth apps over time. Interestingly, only five quality assurance systems could be identified. Compared to quality assessment tools, these quality assurance systems were somewhat more extensive overall and their items contributed to between five and 13 quality dimensions. For example, the items of the health technology assessment module presented by Moshi et al. (35) contributed to a total of 13 of the 14 quality dimensions in our review.

The descriptions of the 14 dimensions were derived based on the extracted criteria from the quality assessment tools and quality assurance systems. The main aim of this work was to identify relevant quality dimensions in the context of mHealth apps in order to conduct focus groups with patients and expert interviews with other stakeholders. This was done to gain further insights into each dimension and to investigate their relevance. Based on this, a set of criteria for evaluating the provision of mHealth apps will be developed.

In addition to the use within the research project, our findings can also be used as an orientation in the development of an mHealth app or related assessment instruments such as checklists. The dimensions should not be seen as a simple rating tool. Developers and researchers should critically reflect on each quality dimension.

In the following, the application of the quality dimensions “Information and Transparency” as well as “Validity & (Added) Value” will be briefly presented using the example of an mHealth app for diabetes management. In the context of “Information and Transparency”, it should be ensured that the information is presented transparently and that the relevant target group is clearly defined. For example, information should be provided on the responsible manufacturer, the costs involved and how to deal with problems during the use, or what forms of support are generally available. More indication-specific, the quality dimension “Validity & (Added) Value” should ask whether the content and functions are evidence-based and in-line with published guidelines. Recorded vital signs such as blood glucose must be clear, complete, accurate, relevant and useful. The information provided should be supported by (scientific) sources. Endocrinologists and other stakeholders should be involved in the development and evaluation process. The final application should be subject to clinical trials to demonstrate the patient benefit.

In a next step in the QuaSiApps project, these quality assurance systems will be analyzed and checked for their transferability to the German context. Interestingly, some quality assurance systems in particular show a certain degree of flexibility, thereby taking into account the dynamic developments in the mHealth sector. For example, Yasini et al. (90) developed a multidimensional scale that is completed in a web-based, self-administered questionnaire. The resulting report is both app-specific and applicable to all types of mHealth apps.

The appropriateness of the identified 14 dimensions has to be examined from a bottom-up patients' perspective as well as from a top-down healthcare system perspective to develop a quality assurance system feasible for the German health care system. As described, there are common dimensions for the quality assessment of mHealth apps that are included in many of the approaches analyzed, such as usability, data privacy and validity. Concerning the additional dimensions that we found, the question arises as to how they relate to these classic dimensions. The International Organization for Standardization (ISO) defines quality as the “degree to which a set of inherent characteristics […] of an object […] fulfils requirements” (92). It is to be discussed whether quality dimensions such as “cost(-effectiveness)” represent inherent quality characteristics of an object and thus, their suitability needs to be discussed.

As mentioned above, the approaches included in this review differ from traditional quality assurance concepts. A variety of framework concepts for quality assurance in the healthcare sector exist. They are similar to each other, but have different focuses depending on their objectives (e.g., whether they were designed for quality improvement in the healthcare system, to compare the quality of healthcare internationally, as a template for the accreditation of healthcare services, etc.). In Germany, the Institute for Quality Assurance and Transparency in Healthcare (IQTIG) acts as the central scientific institute for quality assurance in the healthcare sector. The framework concept, whose requirements are based on the principles of patient-centeredness, contains the quality dimensions “effectiveness”, “safety”, “responsiveness”, “timeliness”, “appropriateness”, and “coordination and continuity” (93).

The BfArM's fast-track procedure includes requirements in its checklists relating to “product safety and functionality”, “privacy and information security”, “interoperability”, “robustness”, “consumer protection”, “usability”, “provider support”, “medical content quality” and “patient safety”. Thereby, the fast-track procedure ensures pre-selection by including criteria which are also included in many of the quality assessment tools and quality assurance systems identified in this review. The next step in the QuaSiApps project will be to analyze the transferability of results obtained in this scoping review into a concept for continuous quality assurance of DiGAs, also against the criteria already used in the fast-track procedure. QuaSiApps includes literature reviews, focus groups with users and patients, and interviews with health care stakeholders. Based on the results, proposals for procedural purposes and quality dimensions will be formulated. These will be agreed and refined in expert workshops. The project aims to develop a set of quality aspects and corresponding quality characteristics, quality requirements, quality indicators and measurement tools.

While we are not aware of a literature review specifically on quality assurance systems, at the time of our search several literature reviews on the quality assessment of mHealth apps had already been published (91, 94–99). The most recent review of these included literature published up to December 2022 (99). The authors identified a set of 216 evaluation criteria and 6 relevant dimensions (“context”, “stakeholder involvement”, “development process”, “evaluation”, “implementation”, and “features and requirements”). Although the systemization of the dimensions differs from ours, the content is comparable. This could be indicative for the relevance of the findings.

For example, Azad-Khaneghah et al. (94) conducted a systematic review to identify rating scales used to evaluate usability and quality of mHealth apps. They note that the identified scales ask about different criteria and it is therefore unclear whether the scales actually measure the same construct. Similar to our review, a theoretical basis for the construct of app quality could only be identified to a very limited extent, which is also reflected by the lack of definition of the term “quality” in the included literature. Similarly, McKay et al. (96), who conducted a systematic review of evaluation approaches for apps in the area of health behavior change, criticize the incompleteness of the evaluation criteria, resulting in the authors being unable to propose a uniform best-practice approach to the evaluation of mHealth apps.

Transferring one of the identified systems without adapting to the German healthcare system would not be appropriate. Therefore, further steps are necessary to develop a quality assurance system operating on the system level. The 14 dimensions identified need to be further explored to determine whether they address the potential risks to the quality of health care, and they need to be reflected by stakeholders in the German health care system. Our review has a number of limitations. We searched three databases and also included literature from the field of psychology by searching PsycInfo, but it cannot be ruled out that a more extensive search might have led to additional results. In addition, the exclusive use of bibliometric databases and the omission of secondary literature are limitations in this context. With regard to the search strategy, there is currently disagreement on terminology (27). Therefore, different strategies were tested beforehand and the results were compared to ensure an optimal search strategy. A further potential limitation arises from the inclusion of literature published between January 1st, 2016 and July 26th, 2021. Since we also included older quality assessment tools and quality assurance systems via application studies published during this period, relevant instruments developed before 2016, such as the SUS (20), the NPS (86), or the Silberg Scale (73) were also covered. Our review only included articles up to mid-2021. However, a current review pointed out older assessment tools such as the SUS (1996) (20), the MARS (2015) (18), the PSSUQ (1995) (85), and the uMARS (2016) (63) are still among the most commonly used in mHealth assessment (99). Therefore, we are confident that our search strategy has enabled us to include a large proportion of quality assessment tools and quality assurance systems currently in use.

In addition, we only included articles in German and English language. It is notable that the majority of the quality assessment tools and quality assurance systems included are from English-speaking countries, which may indicate that some tools published in other languages may not have been identified. Nevertheless, we limited our search to articles in bibliographic databases, most of which are published in English. Further, descriptions for quality dimensions were formulated based on the information contained in the quality assessment tools and quality assurance systems. Thus, these descriptions are based on the subjective perception of the researchers and are not based on existing definitions. One reason for choosing this approach was the lack of international agreement on the underlying concepts. As described by Nouri et al. (97), there are major differences in the classification and definition of the individual criteria, so that usability, for example, has very different subcategories depending on the scale or is even seen as a subcategory of functionality, as in Stoyanov et al. (18). The ISO defines usability as the “extent to which a system can be used by specified users to achieve specified goals with effectiveness, efficiency and satisfaction in a specified context of use” (100). While this definition identifies the fundamentals of usability and makes clear that effectiveness, efficiency and satisfaction are key criteria (101), ISO 9241-11:2018 is not intended to describe usability evaluation methods (100). Finally, the study protocol (27) announced the assessment of the suitability of the criteria and derived dimensions for the continuous quality assurance of mHealth apps in Germany as part of this review. In the light of our results, this seems unattainable without taking further steps (e.g., focus groups with patients). Results will be published elsewhere.

Concluding, this review serves as a building block of a continuous quality assurance system for mHealth apps in Germany. Based on our findings, we agree with Nouri et al. (97) that it is challenging to define suitable evaluation criteria for the wide range of functionalities and application areas of apps. In addition, apps are constantly evolving, which means that quality assessment tools and quality assurance systems will also need to constantly adapt.

## Data Availability

The original contributions presented in the study are included in the article/**Supplementary Material**, further inquiries can be directed to the corresponding author.
