# Supplementary material for: Quality assessment of mHealth apps: a scoping review
Source: Front Health Serv. 2024 May 1;4:1372871. doi: 10.3389/frhs.2024.1372871 (PMC11094264; doi:10.3389/frhs.2024.1372871)
Supplement: Supplementary file 1 [file Table1.docx]

**Appendix A:** List of studies excluded in full-text screening.

| **#** | **Reference** | **Title** | **Reason for exclusion** |
| --- | --- | --- | --- |
| 1 | Albrecht 2018 | [Assessing the benefits of digital health solutions in the societal reimbursement context] | No concept of quality assessment/assurance |
| 2 | Biviji 2019 | Evaluating the quality of mobile health apps for Maternal and Child Health (MCH) | Not disease-independent |
| 3 | Brahmbhatt 2017 | Diabetes mHealth Apps: Designing for Greater Uptake | No concept of quality assessment/assurance |
| 4 | Brinkel 2017 | Mobile phone-based interactive voice response as a tool for improving access to healthcare in remote areas in Ghana – an evaluation of user experiences | No DHA |
| 5 | Bruce 2020 | Evaluating Patient-Centered Mobile Health Technologies: Definitions, Methodologies, and Outcomes | Type of publication |
| 6 | Bry 2018 | Consumer Smartphone Apps Marketed for Child and Adolescent Anxiety: A Systematic Review and Content Analysis | No concept of quality assessment/assurance |
| 7 | Buechi 2017 | Evidence assessing the diagnostic performance of medical smartphone apps: A systematic review and exploratory meta-analysis | No concept of quality assessment/assurance |
| 8 | Chaniaud 2020 | Effect of Prior Health Knowledge on the Usability of Two Home Medical Devices: Usability Study | No DHA |
| 9 | Cheng 2020 | Content and Quality of Infant Feeding Smartphone Apps: Five-Year Update on a Systematic Search and Evaluation | Not outpatient |
| 10 | Chumkasian 2021 | Adaptation of the MAUQ and usability evaluation of a mobile phone–based system to promote eye donation | No DHA |
| 11 | Denecke 2020 | How to Evaluate Health Applications with Conversational User Interface? | No DHA |
| 12 | Dewar 2017 | Developing a measure of engagement with telehealth systems: The mHealth Technology Engagement Index | No concept of quality assessment/assurance |
| 13 | Fedele 2019 | Topical Review: Design Considerations When Creating Pediatric Mobile Health Interventions: Applying the IDEAS Framework | Type of publication |
| 14 | Fijačko 2020 | The Effects of Gamification and Oral Self-Care on Oral Hygiene in Children: Systematic Search in App Stores and Evaluation of Apps | Not outpatient |
| 15 | Fraynt 2018 | An evaluation of mobile applications designed to assist service members and veterans transitioning to civilian life | No DHA |
| 16 | Gasteiger 2021 | Sticky apps, not sticky hands: A systematic review and content synthesis of hand hygiene mobile apps | Not disease-independent |
| 17 | Guo 2017 | Assessing the Quality of Mobile Exercise Apps Based on the American College of Sports Medicine Guidelines: A Reliable and Valid Scoring Instrument | Not disease-independent |
| 18 | Ha 2016 | Evaluation of a Mobile Health Approach to Tuberculosis Contact Tracing in Botswana | Not outpatient |
| 19 | Heiney 2020 | A smartphone app for self-management of heart failure in older African Americans: Feasibility and usability study | No concept of quality assessment/assurance |
| 20 | Herbuela 2021 | Early Detection of Dengue Fever Outbreaks Using a Surveillance App (Mozzify): Cross-sectional Mixed Methods Usability Study | Not outpatient |
| 21 | Hochstenbach 2016 | Feasibility of a mobile and web-based intervention to support self-management in outpatients with cancer pain | Not disease-independent |
| 22 | Hsia 2020 | Developing and evaluating ASTHMAXcel adventures: A novel gamified mobile application for pediatric patients with asthma | No DHA |
| 23 | Huberty 2020 | Cancer patients’ and survivors’ perceptions of the calm app: Cross-sectional descriptive study | Not disease-independent |
| 24 | Jones 2020 | Quality of Psychoeducational Apps for Military Members With Mild Traumatic Brain Injury: An Evaluation Utilizing the Mobile Application Rating Scale | No DHA |
| 25 | Kaczmarczyk 2021 | Oral health promotion apps: An assessment of message and behaviour change potential | Not outpatient |
| 26 | Kahnbach 2021 | Quality and adoption of COVID-19 tracing apps and recommendations for development: Systematic interdisciplinary review of European apps | Not outpatient |
| 27 | Kawakyu 2019 | Development and Implementation of a Mobile Phone-Based Prevention of Mother-To-Child Transmission of HIV Cascade Analysis Tool: Usability and Feasibility Testing in Kenya and Mozambique | Not for patient use |
| 28 | Keikhosrokiani 2020 | Assessment of a medical information system: The mediating role of use and user satisfaction on the success of human interaction with the mobile healthcare system (iHeart) | No concept of quality assessment/assurance |
| 29 | Khowaja 2020 | New Checklist for the Heuristic Evaluation of mHealth Apps (HE4EH): Development and Usability Study | Not disease-independent |
| 30 | Kramer 2017 | Value of Health Apps?: How to Assess Quality and Risk? Which Decision Aids Provide Guidance? | Type of publication |
| 31 | Kruse 2020 | Measures of Effectiveness, Efficiency, and Quality of Telemedicine in the Management of Alcohol Abuse, Addiction, and Rehabilitation: Systematic Review | No concept of quality assessment/assurance |
| 32 | Lagan 2021 | Mental Health App Evaluation: Updating the American Psychiatric Association's Framework Through a Stakeholder-Engaged Workshop | Type of publication |
| 33 | Lan 2018 | Review of cognitive behavioural therapy mobile apps using a reference architecture embedded in the patient-provider relationship | Not disease-independent |
| 34 | Langlet 2020 | Formative Evaluation of a Smartphone App for Monitoring Daily Meal Distribution and Food Selection in Adolescents: Acceptability and Usability Study | Not outpatient |
| 35 | Larbi 2020 | Methods and evaluation criteria for apps and digital interventions for diabetes self-management: Systematic review | Not disease-independent |
| 36 | Lee 2017 | Use of a Mobile Application for Self-Monitoring Dietary Intake: Feasibility Test and an Intervention Study | Not outpatient |
| 37 | Loy 2016 | Quality assessment of medical apps that target medication-related problems | Not disease-independent |
| 38 | Luna-Perejon 2019 | Evaluation of user satisfaction and usability of a mobile app for smoking cessation | No concept of quality assessment/assurance |
| 39 | Macis 2020 | Design and Usability Assessment of a Multi-Device SOA-Based Telecare Framework for the Elderly | No DHA |
| 40 | Messner 2019 | mHealth applications: Potentials, limitations, current quality and future directions | Type of publication |
| 41 | Moglia 2016 | Evaluation of Smartphone Menstrual Cycle Tracking Applications Using an Adapted APPLICATIONS Scoring System | Not outpatient |
| 42 | Morse 2018 | Mobile Health Applications for Pediatric Care: Review and Comparison | No concept of quality assessment/assurance |
| 43 | Moura 2020 | Assessing Access Control Risk for mHealth: A Delphi Study to Categorize Security of Health Data and Provide Risk Assessment for Mobile Apps | No concept of quality assessment/assurance |
| 44 | Paganini 2021 | Quality of Physical Activity Apps: Systematic Search in App Stores and Content Analysis | Not outpatient |
| 45 | Pecorelli 2018 | An app for patient education and self-audit within an enhanced recovery program for bowel surgery: a pilot study assessing validity and usability | Not outpatient |
| 46 | Portelli 2016 | A quality review of smartphone applications for the management of pain | Not disease-independent |
| 47 | Radcliffe 2021 | A Pilot Evaluation of mHealth App Accessibility for Three Top-Rated Weight Management Apps by People with Disabilities | Not outpatient |
| 48 | Rodrigues 2021 | Mobile Applications (Apps) to Support the Hepatitis C Treatment: A Systematic Search in App Stores | Not for patient use |
| 49 | Ruggiano 2021 | Chatbots to support people with dementia and their caregivers: Systematic review of functions and quality | No DHA |
| 50 | Schuman-Olivier 2018 | MySafeRx: a mobile technology platform integrating motivational coaching, adherence monitoring, and electronic pill dispensing for enhancing buprenorphine/naloxone adherence during opioid use disorder treatment: a pilot study | No DHA |
| 51 | Seidman 2019 | Feasibility and Acceptance Testing of a Mobile Application Providing Psychosocial Support for Parents of Children and Adolescents With Chronic Pain: Results of a Nonrandomized Trial | No concept of quality assessment/assurance |
| 52 | Singh 2016 | Developing a Framework for Evaluating the Patient Engagement, Quality, and Safety of Mobile Health Applications | Type of publication |
| 53 | Sobrinho 2018 | Design and evaluation of a mobile application to assist the self-monitoring of the chronic kidney disease in developing countries | No concept of quality assessment/assurance |
| 54 | Stec 2019 | Client-Centered Mobile Health Care Applications: Using the Mobile Application Rating Scale Instrument for Evidence-Based Evaluation | Type of publication |
| 55 | Sucala 2017 | Anxiety: There is an app for that. A systematic review of anxiety apps | No concept of quality assessment/assurance |
| 56 | Sun 2020 | A Sexual Health Promotion App for Transgender Women (Trans Women Connected): Development and Usability Study | No concept of quality assessment/assurance |
| 57 | Sung 2020 | Developing a Mobile App for Monitoring Medical Record Changes Using Blockchain: Development and Usability Study | Not outpatient |
| 58 | Symsack 2021 | Usability Assessment of the Rehabilitation Lower-limb Orthopedic Assistive Device by Service Members and Veterans With Lower Limb Loss | Not disease-independent |
| 59 | Torous 2016 | Quality assessment of self-directed software and mobile applications for the treatment of mental illness | Type of publication |
| 60 | Vallespin 2016 | Ensuring Evidence-Based Safe and Effective mHealth Applications | Not disease-independent |
| 61 | van Rosmalen-Nooijens 2017 | Young People, Adult Worries: Randomized Controlled Trial and Feasibility Study of the Internet-Based Self-Support Method Feel the ViBe" for Adolescents and Young Adults Exposed to Family Violence" | No concept of quality assessment/assurance |
| 62 | Whittington 2020 | Detecting physical abilities through smartphone sensors: an assistive technology application | No DHA |
| 63 | Wyatt 2018 | How can clinicians, specialty societies and others evaluate and improve the quality of apps for patient use? | Type of publication |
| 64 | Zare Moayedi 2018 | Developing an Android-Based Patient Decision Aid Based on Ottawa Standards for Patients After Kidney Transplant and Its Usability Evaluation | Not outpatient |
| 65 | Zhou 2019 | Applying a User-Centered Approach to Building a Mobile Personal Health Record App: Development and Usability Study | Not outpatient |

DHA: Digital health application: HCP: Healthcare professional.
