# Supplementary material for: Quality assessment of mHealth apps: a scoping review
Source: Front Health Serv. 2024 May 1;4:1372871. doi: 10.3389/frhs.2024.1372871 (PMC11094264; doi:10.3389/frhs.2024.1372871)
Supplement: Supplementary file 2 [file Table2.docx]

**Appendix B:** Overview of included studies.

1. *Application studies*
   1. MARS / MARS-G / uMARS / modified MARS

| **No.** | **Author(s)** | **Year** | **Country** | **Quality Assessment Tool** | **Disease(s) / Field(s) of application** | **Number of investigated apps** | **Intervention** | **Study type** | **Origin** |
| --- | --- | --- | --- | --- | --- | --- | --- | --- | --- |
| 1 | Alhuwail et al. | 2020 | Kuwait | MARS | Depression and anxiety | 23 apps | A systematic search of smartphone apps (in Arabic-language) was conducted in the Apple App and the Google Play Stores. At least three researchers used the MARS to assess the apps. | App Review | MARS à Stoyanov et al. (2015) |
| 2 | Amor-García et al. | 2020 | Spain | MARS | Genitourinary cancers | 46 apps | A search for all available apps for patients with genitourinary cancers was conducted for iOS and Android platforms. Two independent researchers used the MARS to assess the apps. | App Review | MARS à Stoyanov et al. (2015) |
| 3 | Choi et al. | 2018 | USA | MARS, IMS functionality scores | Sleep self-management | 73 apps | A systematic search was conducted in four mobile app stores (iTunes Appstore, Android Google Play, Amazon Appstore and Microsoft Appstore). Two reviewers were trained in the use of MARS and IMS functionality score and assessed the apps. | App Review | MARS à Stoyanov et al. (2015)  IMS functionality scores à Aitken et al. (2013) |
| 4 | Choi et al. | 2020 | USA | uMARS | Alzheimer’s disease or related dementias | 36 apps | A review of (free, English-language) apps was conducted in the U.S. Google Play Store (Android) and the U.S. App Store (iOS) was conducted. The quality was evaluated by two reviewers using the uMARS, the end user version of the original MARS. | App Review | uMARS à Stoyanov et al. (2016) |
| 5 | Davalbhatka et al. | 2020 | India, UK, USA | MARS | COVID-19 | 63 apps | A systematic review of the literature and mobile platforms (Google play store and Apple App store) was conducted to assess mobile applications using the MARS. | App Review | MARS à Stoyanov et al. (2015) |
| 6 | Escoffery et al. | 2018 | USA | Modified MARS (4 MARS subcscales + an assessment of satisfaction) | Epilepsy self-management | 20 apps | A review was conducted in the Apple App Store. English-language apps costing less than $5.00 were assessed by two reviewers using the MARS. | App Review | MARS à Stoyanov et al. (2015) |
| 7 | Fuller-Tyszkiewicz et al. | 2020 | Australia | Modified MARS (Excluding the items assessing the entertainment value and evidence base for the app) | Reducing stress and improving well-being of people providing care to family or friends with a physical or mental disability | 1 app | A parallel, single-blind, randomized controlled trial was conducted. 138 participants (Australian, > 18 years of age, fluent in English, helping to support a friend or relative with a physical or mental condition/disability, able to access an Apple iOS mobile phone device with internet access, and not have participated in an eHealth intervention within the previous 6 months) were recruited online and separated in an intervention group (n = 68) and active control group (n = 100). Both apps included self-monitoring functions and feedback but only the intervention group had intervention modules included.  Participants used the assigned app for 5 weeks with weekly contact from the research team. Within a postintervention assessment, the intervention group assessed the app quality using the modified MARS.  A follow-up survey was conducted 4 months after completing the postintervention. | Randomized Controlled Trial | MARS à Stoyanov et al. (2015) |
| 8 | Gong et al. | 2020 | China (Authors: Australia, China, UK) | MARS, IMS functionality scores | Diabetes self-management | 67 apps | A systematic search was conducted by two reviewers in four Chinese app stores. the Apple iTunes Store, Tencent Myapp, 360 Mobile Assistand and Baidu Mobile Assistant. Included app were downloaded, screened and independently tested by a team of five raters. App quality was assessed using the MARS. | App Review | MARS à Stoyanov et al. (2015)  IMS functionality scores à Aitken et al. (2013) |
| 9 | Liu et al. | 2021 | China | MARS + Best Practices: Mobile App Privacy & Security | Traditional Chinese Medicine and modern medicine in China | 658 apps (Thereof, 81 apps for quality assessment) | A systematic search was conducted to identify Chinese-language traditional Chinese medicine and modern medicine apps in Apple iTunes app store, Tencent Myapp, Oppo and Huawei.  After classification in either traditional Chinese medicine and modern medicine apps all apps were downloaded and assessed by two reviewers using a standard data extraction form.  Quality was assessed by two independent reviewers using the MARS for the top 25 Android and top 25 iOS apps from traditional Chinese medicine and modern medicine respectively. | App Review | MARS à Stoyanov et al. (2015)  Best Practices: Mobile App Privacy & Security à Internet Society (2014) |
| 10 | Mehdi et al. | 2020 | Germany / Switzerland (Authors) | MARS | Tinnitus | 34 apps | Google Play Store and Apple App Store were systematically searched for apps with English title and description focusing on tinnitus Cognitive behavioural therapy, or self-help as the main subject.  Identified apps were assessed on quality by 4 raters using the MARS | App Review | MARS à Stoyanov et al. (2015) |
| 11 | Muntaner-Mas et al. | 2019 | Spain / USA / Canada / Sweden (Authors) | MARS + developed standardized instrument | Fitness / Cardiorespiratory fitness | 88 apps | Scientific literature as well as available fitness apps (in Google Play and the App Store) were systematically reviewed.  Selected apps were downloaded and used by two reviewers. Subsequently qualitative assessments were carried out using the MARS to rate the app quality and a standardized developed instrument to evaluate some features of the apps. | App-Review & Systematic Literature Review | MARS à Stoyanov et al. (2015) |
| 12 | Myers et al. | 2020 | USA | MARS | Depression | 31 apps | A systematic review of mobile apps was conducted in Android Google Play Store, Apple App Store, and the Amazon Appstore.  Included apps were reviewed at least by two reviewers using the MARS, IMS Institute for Healthcare Informatics functionality score, and six specific depression self-management features. | App Review | MARS à Stoyanov et al. (2015) |
| 13 | Nguyen et al. | 2021 | Canada | MARS | Diabetes | 75 apps | Apps were searched in the Android Play Store and iOS App Store, screened and evaluated by two reviewers. Quality was assessed using the MARS. | App Review | MARS à Stoyanov et al. (2015) |
| 14 | Pearsons et al. | 2021 | UK, Australia (Authors) | MARS + Best Practices: Privacy | Atrial fibrillation | 5 apps | Google Play and Apple App Store were searched for (paid and free) English-language apps.  Six reviewers used the MARS to assess overall app quality. | App Review | MARS à Stoyanov et al. (2015)  Best Practices: Privacy à Internet Society (2019) |
| 15 | Sereda et al. | 2019 | UK | MARS | Tinnitus | 55 apps (Thereof, 18 apps for quality assessment) | 643 participants took place in a web-based survey consisting of 33 open and closed questions. 120 respondents listed 55 apps they have tried to manage their tinnitus. 18 apps which were listed by at least two participants were considered for quality assessment. Subsequently, three researchers used the MARS to evaluate the quality of those 18 cited apps. | User survey & quality assessment of most named apps | MARS à Stoyanov et al. (2015) |
| 16 | Shang et al. | 2019 | China | MARS | Mental health | 63 apps | A search was conducted in the top three Chinese Android app markets (Baidu Mobile Assistant, Tencent MyApp, and 360 Mobile Assistant) and the iOS App Store to identify Chinese-language apps. Identified apps were rated by two independent reviewers using the MARS. | App Review | MARS à Stoyanov et al. (2015) |
| 17 | Tan et al. | 2020 | Australia (Authors: Australia, Portugal, France) | MARS + Accessibility | Allergic Rhinitis and/or asthma | 16 apps | Free of charge, English-language apps were systematically searched in Australian Apple App Store and Android Google Play stores. Assessment was conducted in two stages. First Accessibility (Domain 1), second self-management principle (Domain 2) and usability (Domain 3) were assessed. The usability was independently assessed by four researchers using the MARS. Finally an overall ranking was derived from the three domains. | App Review | MARS à Stoyanov et al. (2015)  Accessibility à Tan et al. (2020) |
| 18 | Terhorst et al. | 2018 | Germany | MARS | Depression | 38 apps | A systematic search was conducted in the German Google-Play-Store and iTunes-Store. Identified apps were assessed by two independent reviewers using the MARS. Apps with above-average rating were evaluated by two practicing psychotherapists regarding their practical relevance. | App Review | MARS à Stoyanov et al. (2015) |
| 19 | Terhorst et al | 2021 | Germany | MARS-G | Pain | 218 apps | The European Google Play and Apple App stores were searched by an automated search engine to identify eligible apps. After screening and checking for systematic criteria, content, quality and privacy features were assessed by two independent reviewers using the German MARS (MARS-G). | App Review | MARS à Stoyanov et al. (2015)  (respectively the German version of the MARS, the MARS-G by Messner et al. (2019)) |
| 20 | Wang et al. | 2021 | USA | MARS | Mental health | 16 apps | 16 of the most popular apps (determined by total downloads) were derived from a publication by Carlo et al. (2019) and subsequently analysed. Three reviewers assessed app quality using the MARS. | Analysis of download numbers and data of user activities as well as quality assessment. | MARS à Stoyanov et al. (2015) |

- 1. SUS

| **No.** | **Author(s)** | **Year** | **Country** | **Quality Assessment Tool** | **Disease(s) / Field(s) of application** | **Number of investigated apps** | **Intervention** | **Study type** | **Origin** |
| --- | --- | --- | --- | --- | --- | --- | --- | --- | --- |
| 21 | de Batlle et al. | 2020 | Spain | SUS + Net Promoter Score (NPS) | Complex chronic patients | 1 app | A three month mHealth-enabled integrated care model was assessed by patients and professionals in terms of acceptability. Patients aged > 55 years, having a hospital admission because of a respiratory or cardiovascular event or having a programmed major elective hip or knee arthroplasty surgery without dementia or cognitive impairment were recruited during an unanticipated admission to the hospital or at the time of surgery.  91 patients were recruited for the intervention arm and 65 for the usual care arm. Thereof, 77 intervention arm and 58 usual care control patients completed the follow-up. 30 health care professionals complemented the results.  After the 3 months integrated care patients’ and staff’s satisfaction with the technology was rated using the Net Promoter Score as well as the SUS. | Assessment of mHealth based, integrated supply | SUS à Brooke (1996)  NPS àReichheld (2003) |
| 22 | Eiring et al. | 2017 | Norway, the UK, and Ukraine (authors: Norway) | SUS | Bipolar disorder | 1 app | A health-optimization system (including a smartphone app and a website) was developed. Patients can invite healthcare practitioners and other collaborators to use the system and contribute according to rights granted by the patient. After iterative redesign summative usability tests were conducted. 19 patients, 11 lay-people, and eight healthcare providers used the SUS to assess the system. | Development and feasibility study | SUS à Brooke (1996) |
| 23 | Grainger et al. | 2020 | New Zealand | SUS | Rheumatoid arthritis | 1 app | A three phases mixed-methods design was used to develop and assess an app. In the first phase interviews were conducted with nine patients and seven healthcare professionals. The second phase served to develop an app and finally the third phase was for evaluating the usability using the SUS. The usability testing was undertaken either with patients using the app on their own medium for one month (n = 16) or with patients using the app once immediately before their routine rheumatologist clinic visit (n = 100). | App development | SUS à Brooke (1996) |
| 24 | Henshall & Davey | 2020 | UK | SUS | Lung cancer survivors (improve symptoms of fatigue, breathlessness and depression) | 1 app | An app aiming to increase exercise activity and improving symptoms was developed. Therefore, a two stage design was used. Stage one served to conduct focus groups with healthcare professionals, patients and family members (n = 21) and stage two to develop iteratively and test the app with lung cancer survivors (n = 6). Usability was assessed using the SUS. | App development | SUS à Brooke (1996) |
| 25 | Hoogeveen et al. | 2018 | Netherlands | SUS | Hepatic glycogen storage disease | 1 app as part of a communication platform | A communication platform was designed using input from software developers, patients, researchers, and healthcare providers. Subsequently, the platform was iteratively developed in two phases. In a third phase the app was implemented in a pilot study of eight patients. Usability was assessed by using the SUS. | App development | SUS à Brooke (1996) |
| 26 | Kizakevich et al. | 2018 | USA | SUS | Stress reduction, sleep improvement, and alcohol moderation | 1 app with additional sensors | An evidence-based app with mindfulness-based relaxation training, behavioural education in sleep quality and alcohol use, and psychometric and psychophysiological data capture for militaries was developed. 31 civilian participants used the app from seven to 14 days. Testing was done iteratively with five participants respectively.  After completion of testing, participants completed an assessment including Likert scale ratings for app features, the comfort and wearability of the sleep monitor and wrist actigraphy. System usability, technical performance, and suggestions for improvements were commented in a common debriefing questionnaire. Finally, the SUS was used to assess the system by four participants of a 14-day and five participants of a 28-day field test. | Mixed-Design study | SUS à Brooke (1996) |
| 27 | Pelle et al. | 2021 | Netherlands | SUS + Backend data analysis | Knee and/or hip osteoarthritis | 1 app | Backend data for the first 26 weeks of use of an RCT intervention group was used to assess use of a Dutch-language e-self-management application. Included patients were aged ≥ 50 years, had to have a smartphone or tablet and were able to read, write and sufficiently communicate in Dutch. Additionally, the system usability was assessed at three and six months using the SUS.  Backend data included: number of logins, number of unique chosen goals, number of unique goals completed, and total number of completed goals. Furthermore, the use of information was quantified by the number of paragraphs read of the educational library. | App use and usability assessment | SUS à Brooke (1996) |
| 28 | Seitz et al. | 2021 | Germany (authors: Germany, Netherlands, UK) | Slightly adjusted SUS | Periodontitis and diabetes | 1 app | A German- and English-language mobile application was developed based on mixed-methods (literature reviews, focus group discussions and a Delphi panel in cooperation with patients, physicians and dentists). Assessment was done by 137 patients (92 patients in general practices and 45 patients in dental practices) aged ≥ 18 years, recruited in the waiting room before their appointment with the doctor, using a slightly adjusted SUS (extended by three questions about the appropriateness of the length of the questionnaire). | App development | SUS à Brooke (1996) |
| 29 | Veazie et al | 2018 | USA | SUS | Diabetes (type 1 and 2) | 11 apps (6 apps for type 1 and 5 apps for type 2 disusabetes) | Literature was searched to identify systematic reviews and technology assessments and recently published studies on commercially available apps. Thereof, relevant apps were identified and related data was collected: available to download on Apple and Android platforms, available devices, cost, privacy/security information, and features. Available apps were additionally rated by three reviewers using the SUS. | App Review | SUS à Brooke (1996) |
| 30 | Wood et al. | 2017 | Australia, USA | SUS + Purpose-developed satisfaction survey | Cystic fibrosis | 1 app | Ten cystic fibrosis patients used the app weekly for four weeks. Subsequently, satisfaction was assessed using a purpose-developed survey which comprised seven items relating to the design and use of the smartphone application and usability was measured with the SUS. | Usability assessment | SUS à Brooke (1996)  Purpose-developed satisfaction survey à Wood et al. (2017) |

- 1. Silberg Scale

| **No.** | **Author(s)** | **Year** | **Country** | **Quality Assessment Tool** | **Disease(s) / Field(s) of application** | **Number of investigated apps** | **Intervention** | **Study type** | **Origin** |
| --- | --- | --- | --- | --- | --- | --- | --- | --- | --- |
| 31 | Xiao et al. | 2016 | China (authors: China, Australia) | Modified Silberg Scale  Twenty Items which are not listed | Cardiovascular diseases | 151 apps | English- and Chinese-language apps were searched in the top six most popular app stores. Subsequently the selected apps were assessed with a seven-dimension quality assessment scale with 20 items derived from the modified Silberg scale and the Technology Acceptance Model. | App Review | Modified Silberg Scale à Silberg et al. (1997) |
| 32 | Zhang et al. | 2017 | Authors: Singapore, Australia, UK | Silberg Scale | Postnatal depression | 14 apps | Apple iTunes as well as Google Android Play store were searched for English-language apps. Subsequently the information quality of the smartphone applications was assessed by three researchers using the Silberg Scale. | App Review | Silberg Scale à Silberg et al. (1997) |

- 1. Other Assessment tools (Questionnaires)

| **No.** | **Author(s)** | **Year** | **Country** | **Quality Assessment Tool** | **Disease(s) / Field(s) of application** | **Number of investigated apps** | **Intervention** | **Study type** | **Origin** |
| --- | --- | --- | --- | --- | --- | --- | --- | --- | --- |
| 33 | Athilingam et al. | 2016 | USA | Self-Confidence questionnaire for providers + MPUQ | Self-management of Heart Failure | 1 app | An app was developed and assessed. The assessment was done by 25 patients and 12 health care workers. After a demonstration of the app and navigating the app for an hour to two hours, patients completed a self-confidence and usability questionnaire and health care workers completed a self-confidence questionnaire. | App assessment | MPUQ à Ryu & Smith-Jackson (2006) |
| 34 | Crosby et al. | 2017 | USA | IBM computer usability satisfaction questionnaires. | Sickle cell disease | 1 app | The study was conducted in three phases. In the first phase 46 patients completed paper surveys of internet access and use. In phase two, 19 patients and eight health care providers identified barriers and co-designed a smartphone app with 5 design experts. Finally, in the third phase five patients completed app feasibility and usability testing using | App development | IBM computer usability satisfaction questionnaires à Lewis (1995) |
| 35 | Fougerouse et al. | 2017 | USA, France | Shorter version of the mHQ | All medical fields | 100 apps | A cross-sectional descriptive study of the top 100 medical applications available in the Google Play store and Apple’s App Store (25 free and 25 paid apps from each store) was conducted. Selected apps were assessed in terms of characteristics using the mHQ. Furthermore, functionalities and relevant medical subject/specialty related to each app was analysed. Consensus was reached by weekly discussion of all working group members. | Cross sectional study | mHQ à Yasini et al. (2016) |
| 36 | Han et al. | 2019 | USA | PEMAT | Pelvic floor exercise | 1 app | 25 patients (female and ≥ 18 years) and 22 providers participated in the study. After using the app for two weeks understandability and actionability was assessed using the PEMAT. | App assessment | PEMAT à Shoemaker et al. (2014) |
| 37 | Huckvale et al. | 2019 | Australia, USA | Specific Privacy Criteria in a Policy | Depression and Smoking Cessation | 36 apps | Android and iOS app marketplaces in the United States and Australia were searched to identify apps. 36 apps (15 Android-only, 14 iOS-only, and 7 available on both platforms) were included. Information about privacy policies and related material with the potential to contain privacy-related content was gathered for each app. Based on a schema of privacy policy quality criteria apps were assessed. | App Review | Specific Privacy Criteria in a Policy à Huckvale et al. (2015) |
| 38 | Turchioe et al. | 2020 | USA | Health-ITUES | Heart failure | 1 app | 168 English- or Spanish-speaking patients with heart failure were recruited from an inpatient cardiac unit and an ambulatory care clinic. After usage of an inclusively designed app, patients assessed usability with the Health-ITUES. | App assessment | Health-ITUES à Schnall et al. (2018) |
| 39 | Sedhom et al. | 2021 | USA | Digital Health Scorecard | Oncology | 22 apps | Google Play and Apple iTunes store were searched for English-language oncology apps with a star rating above 3.5 and more than four reviews. 22 popular mobile health oncology apps were included and assessed using the Digital Health Scorecard. | App assessment | Digital Health Scorecard à Mathews (2019) |

1. *Development studies*
   1. Found in databases
      1. Development studies

| **#** | **Author(s)** | **Year** | **Country** | **Quality Assessment Tool** | **Disease(s) / Field(s) of application** | **Description** | **Quality Dimension (Authors)** | **Quality Dimension (QuaSiApps)** | **Origin / Comments** |
| --- | --- | --- | --- | --- | --- | --- | --- | --- | --- |
| 1 | Berry et al. | 2018 | UK | Mobile Agnew Relationship Measure (mARM) Questionnaire  25 Questions with a seven point Likert Scale (Strongly Disagree – Strongly Agree) | Mental Health | Stage 1: Interviews with nine mental health service users about the concept of therapeutic alliance in the context of a digital health intervention  Stage 2: Rating scales and open-ended questions were modified in terms of replacing “therapist” with the word “app” and presented to 14 service users and 10 mental health staff to assess content and to face validity of the scale.  Stage 3: Findings of the first two stages were used to adapt the measure with the support of a decision-making algorithm about which items to drop, retain, or adapt. | - Digital therapeutic relationship | - Engagement | Agnew Relationship Measure (ARM) |
| 2 | Brown et al. | 2013 | USA | Health IT Usability Evaluation Model (Health-ITUEM) | All fields of health | Two sources of data were used to assess the applicability of the Health-ITUEM for mHealth technology. 10 focus groups were conducted with 70 participants. The topic of the focus groups was on usage of mobile (health) technology. Four of the groups were conducted with participants who have been given a mobile phone with preinstalled health apps 30 days prior to the group setting. | - Error prevention - Completeness - Memorability - Information needs - Flexibility - Learnability - Performance speed - Competency - Other outcomes | - Information & Transparency - Validity & (Added) Value - Engagement - Usability & Design | . |
| 3 | Camacho et al. | 2020 | USA | Technology Evaluation and Assessment Criteria for Health Apps (TEACH-Apps)  Structured 4-phases process to identify eligible apps receptive to diverse local needs, rigorous standards, and current content.  Number of assessment items varies dependent on the process. | All fields of health | A process was developed based on the "Replicating Effective Programs (REP)" framework consisting of four phases: preconditions, preimplementation, implementation, and maintenance and evolution.  Ten stakeholders were recruited to go through the four phases.  Phase 1 "Preconditions": Participants were asked to name apps they know and areas in which apps may be of help. A search for further apps was supplemented. In parallel a committee interested in app evaluation should be recruited. Committee members are responsible for evaluating a certain number of apps and to decide whether they are relevant for the next stage.  Phase 2 "Preimplementation": A committee-meeting is arranged to reflect based on feedback from the preconditions phase what app evaluation criteria should be added or removed from the APA App Evaluation framework. Depending on the selected criteria apps that fail to meet the consumer’s needs can be eliminated. Finally, available apps selected for consideration in the context of the final criteria are assessed. No scores or points are assigned. Only a separation of those apps that have serious flaws from those that may be acceptable is necessary.  Phase 3 "Implementation": All acceptable apps from the preimplementation phase are tested by the committee-members. Feedback on which apps may be a better fit in terms of usability and offer the most clinical value is collected. Finally, an educational handout that explains why the app was selected and pros and cons can be prepared for eligible apps.  Phase 4 "Maintenance and Evolution": At least two times per year the process should be repeated. New apps should be prioritized for evaluation. Overall the volume will be reduced because with further rounds, the apps that meet user needs should rise to the top. | - Privacy - Medical - Price - Ratings - Attributes - Features - Onboarding - Performance | - Information & Transparency - Validity & (Added) Value - (Medical) Safety - Interoperability & Compatibility - Actuality (indirectly process should be conducted twice a year) - Engagement - Data Privacy & Data Security - Usability & Design - Technology - Organizational - Equity & Equality - Cost (-effectiveness) | American Psychiatric Association (APA) App Evaluation framework  Only including examples of questions |
| 4 | Jusob et al. | 2021 | UK | Privacy framework for the management of chronic diseases via mHealth in a post-Covid-19 world  Framework consists out of five layers (including 22 privacy principles, privacy requirements and mechanisms and associated technologies) | Chronic diseases | Based on a modified version of the engineering design process a privacy framework was developed. Therefore, problems were defined and information was gained through literature reviews, analyses of existing regulatory (privacy) frameworks and past research on privacy threats and concerns. Specified requirements were then used to develop the new framework and compare it with existing frameworks.  The developed framework consists of five layers (1) regulatory frameworks for privacy + privacy threats and concerns, (2) privacy framework principles, (3) privacy requirements, (4) mechanisms and associated technologies, (5) prototype | - Privacy | - Validity & (Added) Value - Data Privacy & Data Security | Existing regulatory frameworks |
| 5 | Llorens-Vernet & Miró | 2020 | Spain | Mobile App Development and Assessment Guide (MAG)  Set of 36 criteria for mHealth-related apps | All fields of health | Three information sources were used to develop a mobile app development and assessment guide. First, a systematic review of all pain-related app studies was conducted. Second, websites of professional organizations were searched for health app recommendations. Third, standards governing the development of software for medical devices were investigated on specialized websites of regulatory organizations.  Subsequently, identified criteria were grouped based on their shared characteristics. Finally 18 stakeholders assessed comprehensibility and perceived importance of the resulting criteria. | - Usability - Privacy - Security - Appropriateness and suitability - Transparency and content - Safety - Technical support and updates - Technology | - Information & Transparency - Validity & (Added) Value - (Medical) Safety - Interoperability & Compatibility - Actuality - Data Privacy & Data Security - Usability & Design - Technology - Equity & Equality |  |
| 6 | Minge & Riedel | 2013 | Germany | Ein modularer Fragebogen zur Erfassung des Nutzererlebens (meCUE) | Not restricted to health | Based on widely established and empirically validated model of user experience a modular questionnaire was developed. The construction of the questionnaire as well as the choice of items resulted on the basis of two conducted data collections with respectively 238 subjects. A first validation with 67 persons was successfully conducted. | Product perception:   - Usefulness - Usability - Visual aesthetics - Social identity status - Social identity attachment   User emotions   - Positive emotions - Negative emotions   Consequences   - Product loyalty - Intention to use | - Validity & (Added) Value - Usability & Design - Social | Article is written in German |
| 7 | Moshi et al. | 2020 | Australia | Module for existing health technology assessment (HTA) methodological frameworks to guide the evaluation of mHealth apps.  The module includes 10 different domains with 23 Challenges posed by mHealth apps | All fields of health | Policymakers, healthcare practitioners, and application developers were interviewed to determine possible pathways and impediments to mobile medical applications. Findings were combined with precedent research on mobile medical application reimbursement and regulation to create a module for existing health technology assessment methodological frameworks to guide the evaluation. | - Description and technical characteristics - Current use of the technology - Effectiveness - Safety - Cost-Effectiveness - Organizational aspects - Ethical aspects - Legal aspects - Post-market monitoring - Social aspects | - Information & Transparency - Validity & (Added) Value - (Medical) Safety - Interoperability & Compatibility - Actuality - Data Privacy & Data Security - Usability & Design - Technology - Organizational - Social - Equity & Equality - Cost (-effectiveness) - Legal | Current HTA evaluation frameworks used in Australia |
| 8 | O’Rourke et al. | 2020 | Austria / Germany | Multidimensional App Quality Assessment Tool for Health-Related Apps (AQUA)  Both, the expert and the user version consist of 31 items in seven dimensions. | All fields of health | Questionnaire items were constructed based on existing app-quality assessment tools and guidelines for evaluating health-related app-quality. The items were tested in pretests with six participants who gave qualitative feedback on the items while filling them out. | Expert Version   - Usability - User Engagement - Content - Visual Design - Therapeutic Quality - Security - Information   User Version   - Usability - User Engagement - Content - Visual Design - Therapeutic Quality - Impact - Information | - Information & Transparency - Validity & (Added) Value - (Medical) Safety - Engagement - Data Privacy & Data Security - Usability & Design - Technology | It exists an expert and a user version of the AQUA respectively in English and German |
| 9 | Sadegh et al. | 2018 | Iran | Framework for m-health service development and success evaluation  The proposed framework includes 5 different quality dimensions, different stakeholders and three stages. | All fields of health | Famous frameworks and models in the field of information technology and electronic health were identified from literature. Based on this review and an additional stakeholder analysis an m-health evaluation framework was developed. During the development three major points were considered. 1) Iterative lifecycle and evaluating mHealth services through different stages of lifecycle 2) regarding different dimensions in evaluating different stages of mHealth service lifecycle and 3) effective stakeholders in evaluating different stages of lifecycle.  The framework includes a three-stage life cycle:  Stage 1 "Service Requirement Analysis"  Stage 2 "Service Development”  Stage 3 "Service Delivery" | - Technical - Service Usability - Strategic - Organizational - Social and Legal | - Validity & (Added) Value - (Medical) Safety - Engagement - Usability & Design - Technology - Organizational - Legal |  |

- - 1. Studies including new or self-designed concepts

| **#** | **Author(s)** | **Year** | **Country** | **Quality Assessment Tool** | **Disease(s) / Field(s) of application** | **Description** | **Quality Dimension (Authors)** | **Quality Dimension (QuaSiApps)** | **Origin / Comments** |
| --- | --- | --- | --- | --- | --- | --- | --- | --- | --- |
| 10 | Glattacker et al. | 2020 | Germany | Usability questionnaire  13 items that were based on the System Usability Scale and the Modular Evaluation of Components of User Experience (meCUE) | Allergic Rhinitis | An app evaluation process was conducted. Usability and changes in quality of life, health literacy, and self-efficacy for managing one’s chronic disease were assessed via 2 online surveys. 661 app users took part in the first and 143 in the follow-up survey. | - Simplicity - Functionality - Personal benefit - Design - Access - Overall evaluation | - Validity & (Added) Value - Engagement - Usability & Design - Technology | SUS + Modular Evaluation of Components of User Experience (meCUE) |
| 11 | Huang et al. | 2020 | Singapore, Australia | App-HONcode | Medication Management in Diabetes | The App-HONcode criteria were developed to assess mHealth apps. The initial versions of the criteria were piloted with highly downloaded diabetes apps to refine and improve the clarity of the assessment criteria. 143 apps were assessed against the app assessment criteria. | - Authoritative - Complemen-tarity - Privacy - Attribution - Justifiability - Transparency - Financial disclosure - Advertising policy | - Information & Transparency - Validity & (Added) Value - (Medical) Safety - Data Privacy & Data Security | HONcode principles |
| 12 | Liu et al. | 2021 | China | Assessment of privacy and security in regard to data gathering, sharing, and security.  Seven questions (yes / no) | Traditional Chinese Medicine and Modern Medicine | A systematic search was conducted to identify Chinese-language traditional Chinese medicine and modern medicine apps in Apple iTunes app store, Tencent Myapp, Oppo and Huawei.  After classification in either traditional Chinese medicine and modern medicine apps all apps were downloaded and assessed by two reviewers using a standard data extraction form.  Quality was assessed by two independent reviewers using the MARS for the top 25 Android and top 25 iOS apps from traditional Chinese medicine and modern medicine respectively. | - Privacy and security | - Data Privacy & Data Security - Equity & Equality | Guidance of privacy in mobile apps recommended by the Information Commissioner’s Office + The mobile app privacy and security best practices published by the Online Trust Alliance |
| 13 | Pifarré et al. | 2017 | Spain | Survey to test usability + stability analysis  Eight questions (yes / no / na) | Tobacco-quitting | A free mHealth App for Android was developed and deployed. Its stability was analyzed and usability was assessed by 31 patients. Finally, the economic effect was estimated. | - Usability - Stability | - Engagement - Usability & Design |  |
| 14 | Tan et al. | 2020 | Australia (Authors: Australia, Portugal, France) | Accessibility | Allergic Rhinitis and/or asthma | Free of charge, English-language apps were systematically searched in Australian Apple App Store and Android Google Play stores. Assessment was conducted in two stages. First Accessibility (Domain 1), second self-management principle (Domain 2) and usability (Domain 3) were assessed. The usability was independently assessed by four researchers using the MARS. Finally an overall ranking was derived from the three domains. | - Accessibility | - Interoperability & Compatibility - Actuality |  |
| 15 | Wood et al. | 2017 | Australia / USA | Purpose-developed satisfaction survey  Seven items (5-points Likert-Scale Strongly agree – Strongly Disagree) | Cystic fibrosis | Ten cystic fibrosis patients used the app weekly for four weeks. Subsequently, satisfaction was assessed using a purpose-developed survey which comprised seven items relating to the design and use of the smartphone application and usability was measured with the SUS. | - Satisfaction | - Validity & (Added) Value - Usability & Design - Social |  |

- 1. Found in application or validation studies

| **#** | **Author(s)** | **Year** | **Country** | **Quality Assessment Tool** | **Disease(s) / Field(s) of application** | **Description** | **Quality Dimension (Authors)** | **Quality Dimension (QuaSiApps)** | **Origin / Comments** |
| --- | --- | --- | --- | --- | --- | --- | --- | --- | --- |
| 16 | Baumel et al. | 2017 | USA | ENLIGHT | All fields of health | A systematic review was conducted to identify relevant quality rating criteria. Based on the identified and categorized items the Enlight assessment tool was created. Subsequently the tool was tested on 42 mobile apps and 42 web-based programs targeting modifiable behaviours related to medical illness or mental health. | - Usability - Visual Design - User Engagement - Content - Therapeutic Persuasiveness - Therapeutic Alliance - General Subjective Evaluation of Program’s Potential - Credibility - Privacy - Security | - Information & Transparency - Validity & (Added) Value - Actuality - Engagement - Data Privacy & Data Security - Usability & Design - Organizational - Legal |  |
| 17 | Brooke | 1996 | UK | System Usability Scale (SUS)  10 items (5-Point Likert scale Strongly disagree – Strongly agree) | Not restricted to health | 50 potential questionnaire items were assembled and two examples of software systems were selected (one was "really easy to use" and the other one was "almost impossible to use, even for highly technically skilled users". 20 people rated both systems againgst all 50 items on a 5-point scale. Subsequently, the items leading to the most extreme responses were selected. | - Usability | - Engagement - Usability & Design |  |
| 18 | Doak et al. | 1996 | USA | Suitability Assessment of Materials (SAM)  22 factors | All fields of health | The Suitability Assessment of Materials (SAM) was developed and subsequently validated by 172 health care providers from several cultures. Originally the assessment tool was developed for print material and illustrations, but can also be applied for other media. | - Content - Literacy demand - Graphics - Layout and typography - Learning stimulation, motivation - Cultural appropriate-ness | - Information & Transparency - Engagement - Usability & Design - Equity & Equality |  |
| 19 | Huckvale et al. | 2015 | UK, France, Singapore | Coverage of privacy and security-related topics in privacy policies  Four domains with 24 topics | All fields of health | 79 UK NHS Health Apps Library certified apps (price ≤ 50 USD) were assessed. Therefore manual testing and policy review were conducted by two study researchers. | - Privacy | - Actuality - Data Privacy & Data Security - Legal |  |
| 20 | Lewis | 1995 | USA | ASQ, PSSUQ, CSUQ | Not restricted to health | Three IBM questionnaires (After-Scenario Questionnaire (ASQ), Post-Study System Usability Questionnaire (PSSUQ), Computer System Usability Questionnaire (CSUQ)) measuring user satisfaction with computer system usability are discussed and administration and scoring instructions related to the questionnaires are given. | - Usability | - Information & Transparency - Validity & (Added) Value - Engagement - Usability & Design |  |
| 21 | Mathews et al. | 2019 | USA | Digital Health Scorecard | All fields of health | A Digital Health Scorecard including four components (technical, clinical, usability and cost) is developed. The proposed Digital Health Scorecard is based on a hybrid approach that proactively defines requirements and standards for digital health products; transparently discloses and objectively evaluates them, and reports to industry and the public. The Digital Health Scorecard is for pre- and post-market product evaluation. | - Technical - Clinical - Usability - Cost | - Validity & (Added) Value - Interoperability & Compatibility - Data Privacy & Data Security - Usability & Design - Cost  (-effectiveness) |  |
| 22 | Reichheld | 2003 | USA | Net Promoter Score (NPS)  One defined question (10-point Likert Scale (not at all likely - extremely likely)) | Not restricted to health | The author presents his own research regarding customer satisfaction and loyalty in a narrative way. Together with Sarmetrix, a company that develops software to gather and analyze real-time customer feedback, 20 questions on the Loyalty Acid Test were administered to thousands of customers in six industries. Subsequently, purchase histories were obtained for each person and persons were asked to name specific instances in which they had referred someone else to the company in question. Derived of the combination of these data, the question "How likely is it that you would recommend [company X] to a friend or colleague?” was assessed to be the most important. | - Satisfaction | - Engagement |  |
| 23 | Ryu & Smith-Jackson | 2006 | USA | Mobile Phone Usability Questionnaire (MPUQ)  Six factors, 72 items | Not restricted to health | The psychometric quality of usability questionnaire items was determined. Subsequently, evaluation was conducted with approximately 300 participants. | - Usability | - Information & Transparency - Validity & (Added) Value - Interoperability & Compatibility - Engagement - Usability & Design - Technology |  |
| 24 | Schnall et al. | 2018 | USA | Health Information Technology Usability Evaluation Scale (Health-ITUES) | All fields of health | 92 English-speaking patients with HIV, ≥ 18 years used an app for usability testing. After using the app, patients completed a number of surveys including the Health-ITUES. Subsequently, correlation between the Health-ITUES and a widely used and well-validated usability assessment tool were assessed. | - Impact - Perceived usefulness - Perceived ease of use - User control | - Information & Transparency - Validity & (Added) Value - Engagement - Usability & Design - Equity & Equality |  |
| 25 | Shoemaker et al. | 2014 | USA | Patient Education Materials Assessment Tool (PEMAT)  Two categories with 26 items | All fields of health | Different items from existing instruments and guides were assessed by an expert panel. Subsequently, eligible items were compiled and four rounds of reliability testing were conducted and evidence of construct validity was produced with consumers and readability assessments. | - Understand-ability - Actionability | - Information & Transparency - Validity & (Added) Value - Engagement - Usability & Design - Equity & Equality |  |
| 26 | Silberg et al. | 1997 | USA | Silberg Scale  Four core standards | All fields of health | Four core standards to assess medical information on the internet were defined. | - Authorship - Attribution - Disclosure - Currency | - Information & Transparency - Validity & (Added) Value |  |
| 27 | Stoyanov et al. | 2015 | Australia | Mobile App Rating Scale (MARS)  6 Sections with 29 items | All fields of health | Based on a literature search existing criteria for the assessment of app quality were categorized by an expert panel to develop a mobile app rating scale. The scale was piloted using ten apps. Subsequently, interrater reliability was assessed by using the score for 50 further apps. | - Engagement - Functionality - Aesthetics - Information - App subjective quality - App-specific | - Information & Transparency - Validity & (Added) Value - Engagement - Usability & Design - Technology - Cost (-effectiveness) |  |
| 28 | Stoyanov et al. | 2016 | Australia | User Version of the Mobile Application Rating Scale (uMARS)  6 Sections with 26 items | All fields of health | The Mobile App Rating Scale (MARS) was simplified. Based on this, 13 young people piloted it and created the uMARS. Subsequently, 164 young people participated in a randomized controlled trial of a mHealth app to assess internal consistency and test-retest reliability of the uMARS. App ratings were collected using the uMARS at 1, 3, and 6 month follow up. | - Engagement - Functionality - Aesthetics - Information - App subjective quality - Perceived impact | - Validity & (Added) Value - Engagement - Usability & Design - Technology - Cost (-effectiveness) |  |
| 29 | Yasini et al. | 2016 | France | Multidimensional assessment program for rating the quality of mHealth apps and to seal high quality apps by mHQ logo  Three content axis, two technical axes | All fields of health | 18 experts participated in working groups. Based on a previous study, app quality evaluation criteria found in literature and existing national or European legislation, privacy, security standards, and recommendations criteria were discussed in several meetings. A content analysis of the criteria was performed and the guidelines were validated by expert panels.  Subsequently, developed criteria were integrated in a questionnaire and tested by five app developers to test the readability. The questionnaire was then validated by the expert panels.  The final proceeding of app evaluation includes the self-administered questionnaire a usability check and if both are satisfying, the app will be checked for security aspects with help of external services. | - App classification - Medical aspects & content validity - Legal consistency - Mobile medical app distinction - Ethical issues | - Information & Transparency - Validity & (Added) Value - (Medical) Safety - Interoperability & Compatibility - Actuality - Engagement - Data Privacy & Data Security - Usability & Design - Social - Equity & Equality - Legal |  |

1. *Validation studies*

| **#** | **Author(s)** | **Year** | **Country** | **Quality Assessment Tool** | **Disease(s) / Field(s) of application** | **Description** | **Results** | **Study design** | **Origin** |
| --- | --- | --- | --- | --- | --- | --- | --- | --- | --- |
| 1 | Broekhuis et al. | 2019 | Nether-lands | Logging task performance, think aloud and the System Usability Scale (SUS) | Physical activity, smoking cessation | A usability evaluation protocol incorporating all three benchmarking methods (Logging task performance, think aloud and SUS) was deloyed among 36 participants. The participants were divided into three groups using different eHealth technologies (a gamified application for older adults (n=19), an online tele-rehabilitation portal for healthcare professionals (n=9) and a mobile health app for adolescents (n=8)). | The SUS as a stand-alone usability metric for eHealth is not recommended.  Listing usability issues from think aloud protocols is one of the most effective tools to explain the usability for eHealth. | Case studies | SUS à Brooke (1996) |
| 2 | Dawson et al. | 2020 | USA | Suitability assessment of materials (SAM)    A health literacy and usability heuristic evaluation of a mobile consumer health application    Mobile Applications Rating Scale (MARS) | Breastfeeding, smoking cessation, and asthma | Three apps were chosen and used over a seven-day time period. Four existing evaluation tools, checklists, and guidelines were used by team members to assess selected mHealth apps. Thereby strengths, challenges, and potential gaps within used tools were identified. | Found challenges:   - Subjective nature of the results - Time required for evaluation - Lack of emphasis on evidence-based content - Inadequate tool flexibility   Evaluation tools that assess evidence-based content and the ability of the mobile app to securely integrate with other digital technologies involved in patient care would be beneficial. | Literature research,  App evaluation | SAM à Doak et al. (1996)    A health literacy and usability heuristic evaluation of a mobile consumer health application à Monkman & Kushniruk (2013)    MARS à Stoyanov et al. (2015) |
| 3 | Llorens-Vernet & Miró | 2020 | Spain | Mobile App Development and Assessment Guide (MAG) | Not specified | The Delphi method was used to validate the MAG. Participants included health care professionals, developers and final users. The first round of the process included 42 participants, the second round included 24 participants. | 48 criteria were found to be important. Thereof, most were in the categories “privacy”, “security” and “usability”. | Delphi method | MAG à Llorens-Vernet & Miró (2020) |
| 4 | Miró & Llorens-Vernet | 2021 | Spain | Mobile App Development and Assessment Guide (MAG)    Mobile App Rating Scale (MARS) | Chronic health conditions | The four most downloaded health apps for chronic health conditions found in the App Store and Google Play were assessed with the MAG and the MARS in order to study the interrater reliability. Eight reviewers independently evaluated the quality using the two tools. Subsequently, the interrater reliability was calculated. | Few categories of the MAG and MARS demonstrated a high interrater reliability. Overall the interrater reliability was higher in the MAG compared to MARS | App evaluation | MAG à Llorens-Vernet & Miró (2020)    MARS à Stoyanov et al. (2015) |
| 5 | Terhorst et al. | 2020 | Germany, Lebanon, Brazil, Australia, | Mobile App Rating Scale (MARS)    ENLIGHT |  | A validation study was conducted to evaluate the metric quality of the MARS. Therefore, research groups using the MARS were contacted and asked to provide their primary data. Subsequently, all data sets provided were verified, homogenized, and merged into a single data set. | The MARS is suitable for the transparent quality assessment of mHealth apps. | Literature search, Validation study | MARS à Stoyanov et al. (2015)    ENLIGHT à Baumel et al. (2017) |
