# Supplementary material for: Quality assessment of mHealth apps: a scoping review
Source: Front Health Serv. 2024 May 1;4:1372871. doi: 10.3389/frhs.2024.1372871 (PMC11094264; doi:10.3389/frhs.2024.1372871)
Supplement: Supplementary file 3 [file Table3.docx]

**Appendix C:** Described dimensions in included studies

| **#** | **Author(s) + year** | **Information & Transparency** | **Validity & (Added) Value** | **(Medical) Safety** | **Interoperability & Compatibility** | **Actuality** | **Engagement** | **Data Privacy & Data Security** | **Usability & Design** | **Technology** | **Organizational aspects** | **Social aspects** | **Equity & Equality** | **Cost (-effectiveness)** | **Legal aspects** |
| --- | --- | --- | --- | --- | --- | --- | --- | --- | --- | --- | --- | --- | --- | --- | --- |
| 2 | Baumel et al. (2017) | X | X |  |  | X | X | X | X |  | X |  |  |  | X |
| 3 | Berry et al. (2018) |  |  |  |  |  | X |  |  |  |  |  |  |  |  |
| 4 | Brooke et al. (1996) |  |  |  |  |  | X |  | X |  |  |  |  |  |  |
| 5 | Brown et al. (2013) | X | X |  |  |  | X |  | X |  |  |  |  |  |  |
| 6 | Camacho et al. (2020) | X | X | X | X | (X)^1^ | X | X | X | X | X |  | X | X |  |
| 7 | Doak et al. (1996) | X |  |  |  |  | X |  | X |  |  |  | X |  |  |
| 8 | Glattacker et al. (2020) |  | X |  |  |  | X |  | X | X |  |  |  |  |  |
| 9 | Huang et al. (2020) | X | X | X |  |  |  | X |  |  |  |  |  |  |  |
| 10 | Huckvale et al. (2015) |  |  |  |  | X |  | X |  |  |  |  |  |  | X |
| 12 | Jusob et al. (2021) |  | X |  |  |  |  | X |  |  |  |  |  |  |  |
| 14 | Lewis et al. (1995) | X | X |  |  |  | X |  | X |  |  |  |  |  |  |
| 15 | Liu et al. (2021) |  |  |  |  |  |  | X |  |  |  |  | X |  |  |
| 16 | Llorens-Vernet & Miró (2020) | X | X | X | X | X |  | X | X | X |  |  | X |  |  |
| 17 | Mathews et al. (2019) |  | X |  | X |  |  | X | X |  |  |  |  | X |  |
| 18 | Minge & Riedel (2013) |  | X |  |  |  |  |  | X |  |  | X |  |  |  |
| 19 | Moshi et al. (2020) | X | X | X | X | X |  | X | X | X | X | X | X | X | X |
| 21 | O'Rourke et al. (2020) | X | X | X |  |  | X | X | X | X |  |  |  |  |  |
| 22 | Piffaré et al. (2017) |  |  |  |  |  | X |  | X |  |  |  |  |  |  |
| 23 | Reichheld et al. (2003) |  |  |  |  |  | X |  |  |  |  |  |  |  |  |
| 24 | Ryu & Smith-Jackson (2006) | X | X |  | X |  | X |  | X | X |  |  |  |  |  |
| 25 | Sadegh et al. (2018) |  | X | X |  |  | X |  | X | X | X |  |  |  | X |
| 26 | Schnall et al. (2018) | X | X |  |  |  | X |  | X |  |  |  | X |  |  |
| 27 | Schoemaker et al. (2014) | X | X |  |  |  | X |  | X |  |  |  | X |  |  |
| 28 | Silberg et al. (1997) | X | X |  |  |  |  |  |  |  |  |  |  |  |  |
| 29 | Stoyanov et al. (2015) | X | X |  |  |  | X |  | X | X |  |  |  | X |  |
| 30 | Stoyanov et al. (2016) |  | X |  |  |  | X |  | X | X |  |  |  | X |  |
| 31 | Tan et al. (2020) |  |  |  | X | X |  |  |  |  |  |  |  |  |  |
| 32 | Wood et al. (2017) |  | X |  |  |  |  |  | X |  |  | X |  |  |  |
| 33 | Yasini et al. (2016) | X | X | X | X | X | X | X | X |  |  | X | X |  | X |
|  | Total | **15** | **21** | **7** | **7** | **7** | **18** | **11** | **21** | **9** | **4** | **4** | **8** | **5** | **5** |

^1^Indirectly: The evaluation process should be conducted twice a year
