# Supplementary material for: Quality assessment of mHealth apps: a scoping review
Source: Front Health Serv. 2024 May 1;4:1372871. doi: 10.3389/frhs.2024.1372871 (PMC11094264; doi:10.3389/frhs.2024.1372871)
Supplement: Supplementary file 4 [file Table4.docx]

**Appendix D:** Full descriptions of the 14 quality dimensions.

*Information & Transparency*

This quality dimension includes a transparent description of the application and who it is suitable for. It also includes information about the manufacturer, the costs involved and how to deal with problems during use, or what forms of support are generally available. Information on privacy and related policies are assigned to the “Data Privacy & Data Security” dimension.

*Validity & (Added) Value*

Medical/therapeutic content and functions must always be evidence-based. They must be clear, complete, accurate, relevant and useful. Information about the (scientific) sources used must be provided. Experts should be involved in the development and evaluation process. The final application should be subject to clinical trials to demonstrate benefit. Patients' subjective benefit is also part of this dimension.

*(Medical) Safety*

(Medical) Safety describes any precautions to protect users (in the medical sense, exclusive data security). It must be pointed out that the application does not replace a doctor or therapist and a contact or hotline should be available in case of emergency. Other security-relevant aspects, such as risks due to misuse and the possible effects, must be defined on an application-specific basis.

*Interoperability & Compatibility*

Interoperability and compatibility are concerned with the technological context in which the application is used. This includes compatibility with operating systems and running devices, as well as interoperability with other devices that can add functionality to the application.

*Actuality*

The "actuality" quality dimension aims to ensure that the application is up to date in all other dimensions. This means that its content and technology are state of the art. This includes regular reviews of the application, as well as descriptions of updates and the reasons for them. Changes to the functionality of the application should always be communicated. Another aspect is performance monitoring.

*Engagement*

Engagement focuses on patient's involvement in the application. This can be indicated by the extent of use or the intention to use the application long term, and can be strengthened by calls to action, the setting of goals and human attributes such as friendliness, trust, and acceptance. Users can be motivated by interactions, personalization, interesting content and resulting fun during use. In contrast to the intention to use, the subjective benefit is not part of this dimension but belongs to Validity & (Added) Value.

*Data Privacy & Data Security*

This quality dimension aims to ensure secure and transparent handling of data. It should be emphasized that only data necessary for the app should be collected. Users should be informed simply, clearly and transparently about how their data is handled. Data privacy technologies used and their weak points are also part of this dimension.

*Usability & Design*

Usability provides information on how difficult / complex it is to operate and use the app. Usability can be indicated by simplicity of use. Both direct and long-term use should be taken into account. The design includes the presentation and associated clarity. The application itself, but also the results provided, should be clear and concise. Integrated functions should always be fit for purpose. The usability should be tested by usage tests before publication.

*Technology*

All technological aspects which are related to the application or the executing devices (excluding compatibility or data privacy and data security) are assigned to the "Technology" dimension. This includes, in particular, trouble-free use, economical resource consumption and, where applicable, the blocking of distractions. Technological measurement errors or measurement inaccuracies with regard to vital parameters are assigned to the Validity & (Added) Value" dimension.

*Organizational aspects*

This dimension considers the integration of the application into the exisiting care landscape. In particular, this includes the knowledge and skills of service providers and the question of whether devices are available to benefit from the app. In addition to the prerequisites, however, effects on the system are also considered here. These include, for example, effects on the relationship between the person being treated and the treating person, on the treatment itself, or shifted responsibilities.

*Social aspects*

The Social quality dimension includes the aspect of app use in a social context. On the one hand, the influence of the social context on app use is considered, on the other hand, the influence of app use on the social context (exclusive patient-provider relationship). Social influences can be, for example, the exchange with other users or influences on relationships of the patient with other persons (exclusive Health care providers). Through some apps, the independence of the patients can be strengthened.

*Equity & Equality*

Regarding Equity and Equality, equitable access (Accessibility) to mHealth apps should be taken into account. In particular, barriers to access should be kept to a minimum and patients individual needs should be considered. Individual patients should not be excluded from access to mHealth apps on the basis of illness, disability, socio-demographic or socio-economic factors

*Cost (-effectiveness)*

Costs incurred are considered and placed in relation to the benefits resulting from the app. They can be further used for a health technology assessment.

*Legal aspects*

The "Legal" quality dimension describes compliance with existing laws and regulations. The focus here is on issues such as responsibilities, ownership of data and identification as a medical device.
